# Epigenetic activation during T helper 17 cell differentiation is mediated by Tripartite motif containing 28

**DOI:** 10.1038/s41467-018-03852-2

**Published:** 2018-04-12

**Authors:** Yu Jiang, Ying Liu, Huiping Lu, Shao-Cong Sun, Wei Jin, Xiaohu Wang, Chen Dong

**Affiliations:** 1 Institute for Immunology and School of Medicine, Tsinghua University, 100084 Beijing, China; 2 Department of Medicine, Division of Regenerative Medicine, Ansary Stem Cell Institute, Weill Cornell Medical College, 1300 York Avenue, New York, NY 10065 USA; 3 Department of Immunology and Center for Inflammation and Cancer, MD Anderson Cancer Center, Houston, TX 77030 USA; 4 Beijing Key Lab for Immunological Research on Chronic Diseases, 100084 Beijing, China

## Abstract

Epigenetic regulation is important for T-cell fate decision. Although STAT3 is known to initiate Th17 differentiation program, the downstream mechanism is unclear. Here we show that Tripartite motif containing 28 (*Trim28*) expression in Th17 cells is required for Th17-mediated cytokine production and experimental autoimmune diseases. Genome-wide occupancy analysis reveals that TRIM28-bound regions overlap with almost all Th17-specific super-enhancers (SE), and that those SEs are impaired by the deficiency of STAT3 or TRIM28, but not of RORγt. Importantly, IL-6-STAT3 signaling facilitates TRIM28 binding to the *Il17-Il17f* locus, and this process is required for epigenetic activation and high-order chromosomal interaction. TRIM28 also forms a complex with STAT3 and RORγt, and promotes the recruitment of RORγt to its target cytokine genes. Our study thus suggests TRIM28 to be important for the epigenetic activation during Th17 cell differentiation, and prompts the potential use of epigenetic interventions for Th17-related autoimmune diseases.

## Abstract (web summary)

T help 17 (Th17) cells are important mediators for both protective and pathogenic immune reactions, but how their functions are regulated at the epigenetic level is not understood. Here the authors show that TRIM28, a cofactor for transcriptional regulation, is important for epigenetic activation of Th17-related gene loci during Th17 response.

## Introduction

Upon activation, naive CD4^+^ T cells undergo differentiation into different types of T helper cells including T helper 1 (Th1), Th2, Th17 and T follicular helper (Tfh) cells characterized by different cytokine profiles and effector functions. Not only providing a great model for studying gene regulation and cellular differentiation, Th17 cells are also physiologically potent inducers of tissue inflammation with diverse functions in host defense against bacterial and fungal infections and maintenance of barrier homeostasis^1–5^. Transforming growth factor beta (TGF-β) and interleukin 6 (IL-6) are the initial cytokines that drive the lineage commitment of Th17 development^6–8^. Other cytokines like IL-1β, IL-21, and IL-23 are crucial for the expansion, stability, or functional maturation of Th17 cells^9–14^. RAR-related orphan receptor gamma (RORγt) is the master transcription factor for Th17 cells that is necessary and sufficient to drive *Il17* expression and Th17 differentiation, and loss of RORγt in T cells impaired Th17 cell development and resulted in resistance to many experimental inflammatory diseases^15^. RORγt upregulation requires signal transducer and activator of transcription 3 (STAT3), which acts downstream of IL-6 as well as IL-21 and IL-23^14, 16^. STAT3 is also a key factor in inhibiting TGF-β-induced FOXP3 that binds and antagonizes the function of RORγt in Th17 cells, and STAT3 deficiency skews Th17 differentiation towards anti-inflammatory Treg cells^14,16^.

Epigenetic mechanisms have been reported as key players in T-cell differentiation in response to developmental or environmental cues. Global mapping of histone modifications and DNA methylation/demethylation in different T-cell subsets and loss-of-function studies have clearly demonstrated a crucial function of epigenetic regulators in mediating the development and plasticity of T cells^17,18^. Super-enhancers (SE) were discovered as clusters of enhancers in close proximity with unusual strong enrichment of transcription activators. Coactivators, such as p300 and enhancer-related histone modifications like H3K27Ac, have been used to define SEs. Often near genes associated with cell fate determination, SEs exhibit lineage specificity^19^. It has been reported that different T-cell subsets harbor distinct SE catalogs, predominantly at the regions encoding cytokines and related receptors, as measured by p300 loading intensity^20^. Lineage-regulating transcription factors are enriched at SEs; in particular, STAT proteins selectively and preferentially bind to SEs over typical enhancers (TE), and loss of STATs result in impaired p300 recruitment and the establishment of active enhancers in Th1 and Th2 cells^20,21^. Though this suggests STATs function via the SEs, the mechanism underlying SE establishment and regulation remains largely elusive.

Tripartite motif containing 28 (TRIM28), also called TIF1β or KAP1, is a member of the TIF1 family proteins with a PHD domain and a bromo domain in the C-terminal that may recognize specific histone modifications. TRIM28 was initially identified as a nuclear co-repressor for KRAB-domain containing zinc finger proteins^22^. Upon tethering to chromatin by its partners, TRIM28 functions as a scaffold protein to recruit chromatin modifiers like CHD3/Mi2 of the NuRD complex, heterochromatin protein HP1, histone methyltransferase SETDB1 and DNA methyltransferases DNMTs to induce heterochromatin formation, histone deacetylation and DNA methylation that cause gene silencing or imprinting^23–26^. In the immune system, genetic deletion of TRIM28 in all T cells results in autoimmune phenotype and elevated Th17 response in vivo^27^; however, due to the defective function of anti-inflammatory Treg cells in these mice and a lack of molecular study, the intrinsic role of TRIM28 in T helper cell differentiation is unclear.

Here, we identify TRIM28 as an important regulator during Th17 cell development. In contrast to its well-known function as a co-repressor, TRIM28 positively regulates Th17 transcription program, whose deletion results in impaired Th17 differentiation in vitro and ameliorates inflammatory diseases in vivo. Mechanistically, TRIM28 is recruited to STAT3-occupied genes in response to cytokine stimuli, which in turn regulates epigenetic activation, SE establishment, and RORγt recruitment. Thus, TRIM28 is a key factor linking cytokine signaling and epigenetic activation during Th17 cell development.

## Results

### Loss of TRIM28 in naive T cells ameliorates colitis disease

**Fig. 1:**
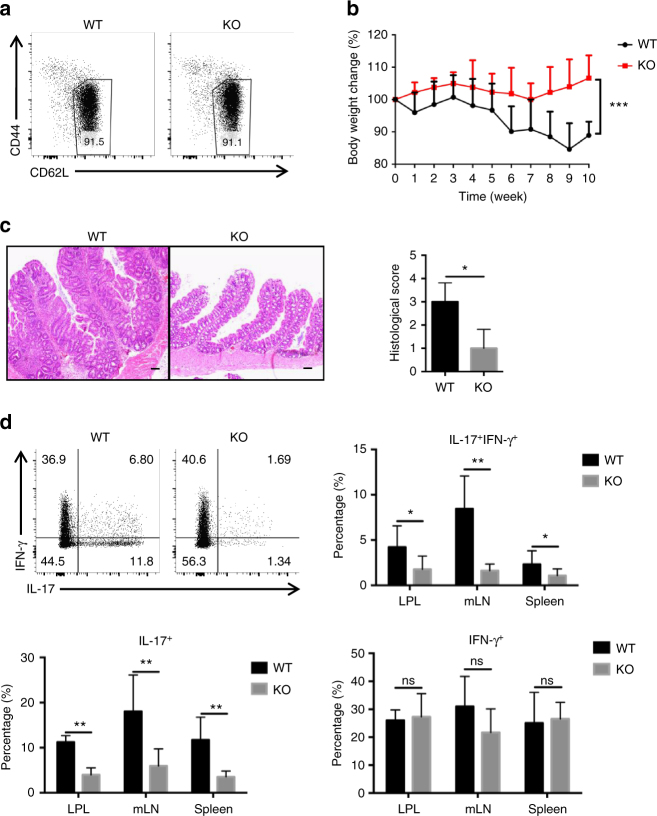
TRIM28 regulated Th17 cell differentiation in vivo. **a** Naive T cells (CD4^+^CD25^−^CD62L^hi^CD44^lo^) isolated from the spleen and peripheral lymph nodes in wild type (WT) and *Trim28*^*−/−*^ mice at the age of 6–7 weeks. **b** Body weight changes in *Rag1*^−^^*/−*^ mice receiving WT or *Trim28*^−^^*/−*^ naive CD4^+^CD25^−^CD62L^hi^ CD45RB^hi^ T cells in the transfer colitis model (WT: *n* = 6, *Trim28*^*−/−*^: *n* = 6, two mice in WT group died before sacrificed), and two-way ANOVA was used for the statistical test. **c** H&E staining of the large intestines of *Rag1*^*−/−*^ mice receiving WT or *Trim28*^*−/−*^ naive T cells (scale bars: 100 μm). **d** Intracellular staining results and statistic data of the IL-17 and IFN-γ expression in the lamina propria (LPL) of large intestine, mesenteric lymph nodes (mLN), and spleen (WT: *n* = 4, *Trim28*^−^^*/−*^: *n* = 6) and Student’s *t *test was used for the statistical test (ns = not significant; *p < 0.05, **p < 0.01, ***p < 0.005). All error bars represent SDs. These experiments were repeated for three times with the consistent results

To investigate the potential role of TRIM28 in helper T-cell function, we generated conditional knockout mice by crossing mice with loxP-flanked *Trim28* alleles to mice with transgenic expression of Cre driven by the *Cd4* promoter (hereafter as *Trim28*^*−/−*^ mice). Examination of peripheral T cells revealed complete excision of the *Trim28* gene in these cells from KO mice. Although it was previously reported that genetic deletion of TRIM28 in T cells using the proximal Lckcre resulted in reduced numbers of T cells in the peripheral and autoimmune phenotypes with elevated IFN-γ and IL-17 production by 23 weeks of age^27^, our *Trim28*^*−/−*^ mice developed apparently normally with no obvious difference in their T-cell development both in the thymus and peripheral (Supplementary Fig. 1a, b). In addition, T activation status in the peripheral appeared normal in these mice at young age (6–8 weeks), and they became slightly more but not significantly activated (*p* > 0.05, by Student’s *t* test) at 20 weeks old (Fig. 1a, Supplementary Fig. 1a, b). Since *Trim28* deletion by Lckcre occurs in the CD4^−^CD8^−^ DN stage, much earlier than CD4cre, and TRIM28 is highly expressed in the thymus^28^, there might be a potential function of TRIM28 in early T-cell development. In addition, higher proportions of naive T cell in our WT mice at 20 weeks of age also suggests different housing environment and microbiota may contribute to the different phenotypes in a cell-extrinsic way.

To assess the function of TRIM28 in naive T cells in vivo, we used a T-cell transfer colitis model in which *Rag1*^*−/−*^ mice were injected with CD4^+^CD45RB^hi^ naive T cells isolated from WT or *Trim28*^*−/−*^ mice. Compared with mice with WT T cells, those containing *Trim28*^*−/−*^ T cells were ameliorated in colitis disease with less body weight loss (Fig. 1b, c). In line with this finding, *Trim28*^*−/−*^ T cells had reduced Th17 cell development in both intestinal lamina propria and mesenteric lymph nodes of recipient mice, while no significant difference (*p* > 0.05, by Student’s *t* test) was found in the total T-cell numbers in lamina propria or IFNγ-producing Th1 or Foxp3^+^ Treg cells (Fig. 1d, Supplementary Fig. 1c).

### TRIM28 regulates Th17 differentiation in vitro

**Fig. 2:**
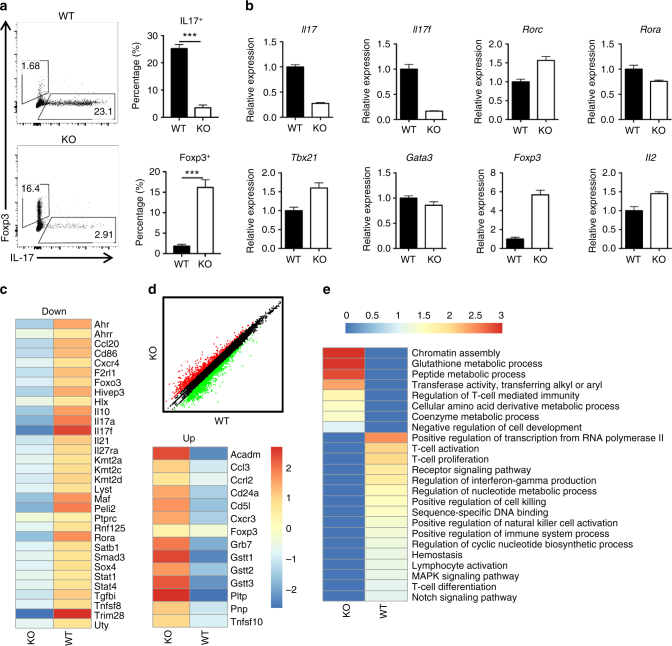
TRIM28 KO CD4^+^ T cells were defective in Th17 differentiation. WT or TRIM28-deficient naive CD4^+^ T cells were polarized into Th17 cells in the presence of TGF-β and IL-6 for 3 days, and then re-stimulated for intracellular staining and mRNA analysis. **a** Intracellular staining results and their statistics (Student’s *t *test; ns not significant; *p < 0.05, **p < 0.01, ***p < 0.005). **b** The relative mRNA expression levels as determined by real-time PCR assay. **c**,** d** Heatmap (left and lower right) and scatter diagram (upper right) of RNA-seq results obtained from WT and TRIM28 KO Th17 cells (anti-CD3 re-stimulated); **e** Heatmap of pathway analysis from RNA-seq data obtained from WT and TRIM28 KO Th17 cells. All error bars represent SDs. **a**, **b** Representative data of three independent experiments

The above results suggest a function of TRIM28 in regulating Th17 cell development. In order to assess this, we isolated naive CD4^+^ T cells from WT and *Trim28*^*−/*^^−^ mice and conducted T-cell differentiation in vitro. In agreement with the in vivo results, *Trim28* ablation led to severely defective Th17 differentiation under all Th17-polarizing conditions examined, characterized by reduced IL-17 and increased Foxp3 expression (Fig. 2a, Supplementary Fig. 2a). However, *Trim28-*deficient cells exhibited no obvious difference in iTreg differentiation, and modest defects in Th1 and Th2 cell development (Supplementary Fig. 2b). Further studies showed that loss of *Trim28* also reduced mRNA levels of the *Il17* and *Il17f* genes, but did not affect key Th17-related transcription factor expression, including *Rorc*, *Stat3*, *Rorα*, *Irf4* and *Batf*, or *Tbx21* and *Gata3*, the master transcription factors for Th1 and Th2 cells, respectively (Fig. 2b, Supplementary Fig. 2c). Notably, *Foxp3* gene expression was increased at both protein and mRNA levels in *Trim28*^−*/*−^ cells in *Trim28*^*−/*^^−^ cells under Th17 conditions (Fig. 2a–c).

To globally analyze the genes regulated by TRIM28, we performed RNA-seq analysis of WT vs. *Trim28*^*−/−*^ Th17 cells generated in vitro in the presence of TGF-β and IL-6. Consistent with the qPCR results, *Trim28* deficiency led to impaired cytokine but not key transcription factor expression under Th17 condition, except increased *Foxp3* expression (Fig. 2b, c). Despite this, we did not observe a global upregulation of other Treg-associated genes, e.g. *Ikzf2*, *Ikzf4*, *Ctla4*, *Gitr*, *Tgfb1*, *Tgfb3*, *Ebi3* in the KO cells (Supplementary Fig. 2c). Interestingly, although TRIM28 was widely regarded as a co-repressor, more genes (652, 67% in all altered genes) were downregulated in the KO cells (Fig. 2d), including many Th17- or immune-related genes (Fig. 2c, e), whereas T-cell-related genes were barely upregulated in the KO cells (Fig. 2c, e), implicating TRIM28 as a positive regulator in Th17 cells.

### TRIM28 intrinsically regulates Th17 cell development

TGF-β induces the expression of both Foxp3 and RORγt, and IL-6 is required for Th17 differentiation in part through inhibition of TGF-β-driven Foxp3 expression^1^. Since loss of *Trim28* did not affect iTreg differentiation but resulted in increased Foxp3 expression in Th17 polarization conditions, it is possible that IL-6 signaling was affected by loss of TRIM28 in Th17 differentiation. To assess this, WT or *Trim28*^*−/*^^−^ naive T cells were polarized into Th17 cells with variable levels of IL-6. Although IL-6 inhibited Foxp3 expression in *Trim28*^*−/−*^ cells in a dose-dependent manner, defective IL-17 expression could not be restored, even at the highest level of IL-6 (Supplementary Fig. 3a). In addition, there was no gross change in the percentages of STAT3 phosphorylation in KO cells during Th17 cell differentiation at different time points (Supplementary Fig. 3b), suggesting that TRIM28 regulates Th17 differentiation independent of the IL-6-STAT3 pathway.

IL-2 is known to constrain Th17 differentiation yet promote Treg generation by signaling through STAT5^29^. However, IL-2 expression was barely affected in the KO T cells (Fig. 2b, Supplementary Fig. 3c), neither was STAT5 phosphorylation (Supplementary Fig. 3b). In addition, though diminishing Foxp3 expression by blocking IL-2, we still observed a significant lower IL-17^+^ percentage in the *Trim28*^*−/−*^ cells (Supplementary Fig. 3d). These above results suggest that TRIM28 might be intrinsically important for Th17 differentiation, independent of Foxp3 suppression, consistent with the impaired Treg cell function after TRIM28 deletion as reported^27^.

**Fig. 3:**
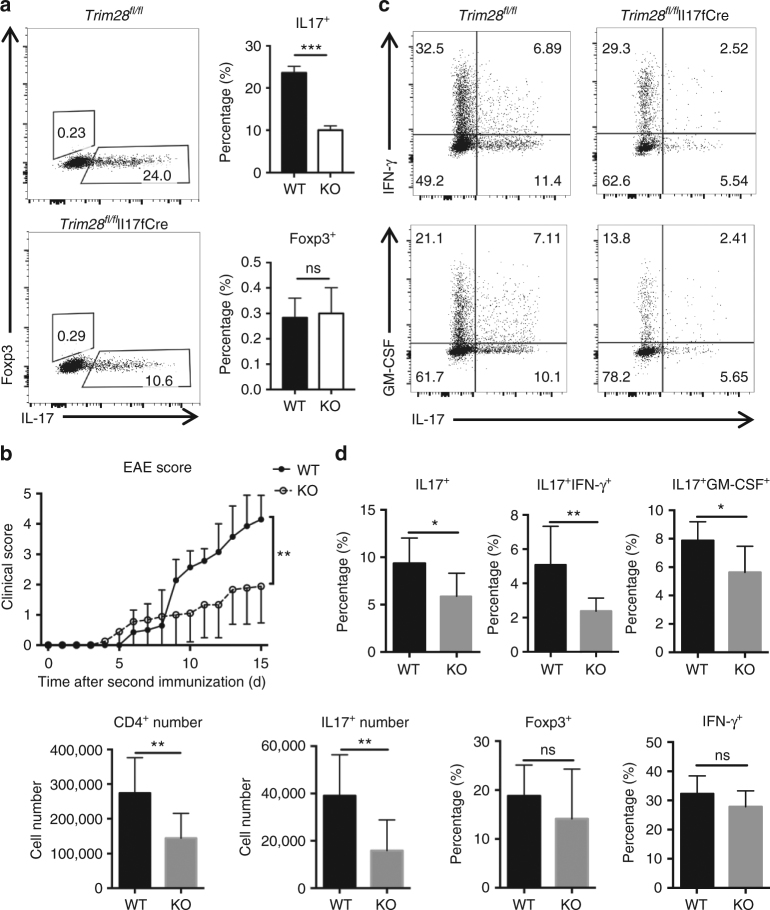
Th17-specific deletion of TRIM28 resulted in defective Th17 differentiation and resistance to EAE. **a** Naive T cells isolated from WT and *Trim28*^*fl/fl*^Il17fcre mice were differentiated into Th17 cells under TGF-β plus IL-6 condition for 3 days, then re-stimulated for cytokine staining, and Student’s *t *test was used for the statistical test. **b** The disease scores of WT and *Trim28*^*fl/fl*^Il17fcre mice in EAE, and two-way ANOVA was used for the statistical test (WT: *n* = 7, *Trim28*^*fl/fl*^Il17fcre: *n* = 7, two mice in WT group died before sacrificed); **c** Intracellular staining of IL-17, GM-CSF and IFN-γ of infiltrated CD4+ T cells in the central nerve system (CNS) of EAE mice. **d** Cellularities and percentages of CNS-infiltrating cells, and Student’s *t *test was used for the statistical test. ns = not significant; *p < 0.05, **p < 0.01, ***p < 0.005. All error bars represent SDs. The data are a representative for three independent experiments

To firmly assess this, TRIM28 was selectively ablated in Th17 cells by crossing *Trim28*^*fl/fl*^ mice with the Il17fcre mice generated in our previous studies^30^. Different from *Cd4*-mediated deletion of *Trim28*, Th17-specific ablation of *Trim28* did not cause increased Foxp3 expression in Th17 cultures, but still caused significant reduced IL-17 production, when compared with the WT cells (Fig. 3a) (*p* < 0.005, by Student’s *t* test). To further confirm this, we utilized the *Trim28*^*fl/fl*^Il17fcre mice to investigate the in vivo function of TRIM28 in Th17-mediated autoimmune disease. In agreement with the in vitro defect, *Trim28*^*fl/fl*^Il17fcre mice developed alleviated EAE diseases (Fig. 3b), with fewer Th17 cells infiltrated into the central nerve system, particularly the IL-17^+^IFN-γ^+^ and IL-17^+^GM-CSF^+^ populations (Fig. 3c, d). Together, our results suggest an essential, intrinsic function of TRIM28 in regulating Th17 cell development both in vitro and in vivo.

### TRIM28 regulates epigenetic activation

**Fig. 4:**
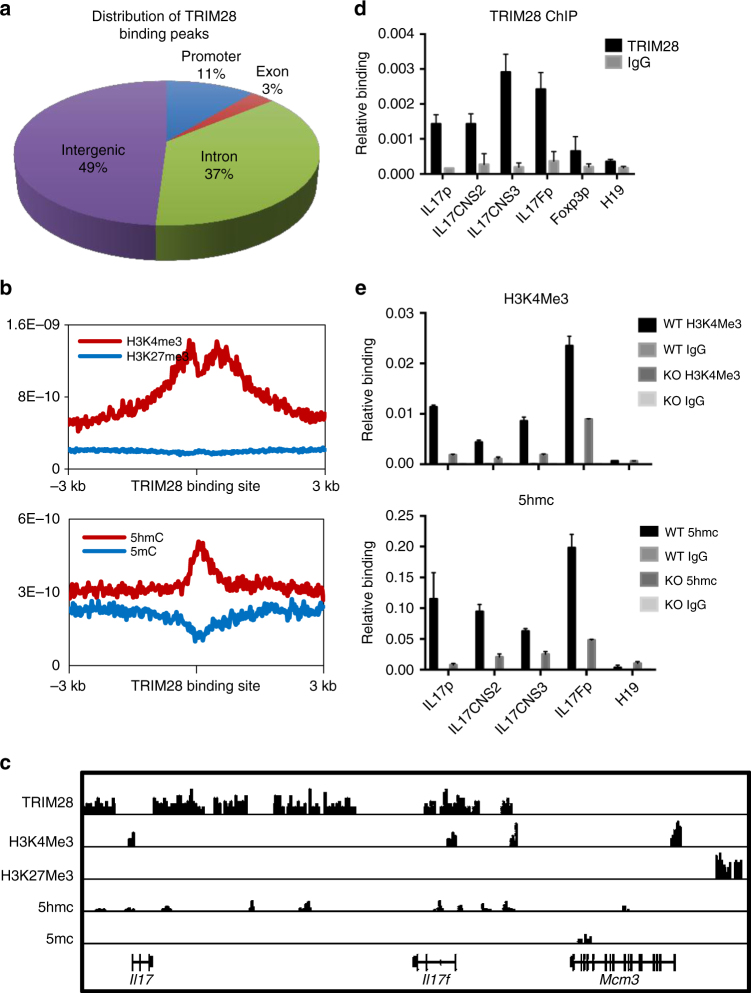
TRIM28 bound to the *Il17-Il17f* gene locus in Th17 cells. **a−d** Naive CD4^+^ T cells were cultured under Th17 condition (TGF-β plus IL-6) for 1 day and then prepared for ChIP-seq or ChIP-qPCR assay using anti-TRIM28 antibody. **a** Distribution of TRIM28-binding peaks in Th17 cells. **b** Alignment of TRIM28 binding peaks with histone modification and DNA methylation/demethylation markers in Th17 cells. **c** IGV browser view of TRIM28 binding peaks with H3K4me3, H3K27me3, 5hmc and 5mc markers at the *Il17-Il17f* gene locus. **d** ChIP-qPCR analysis of TRIM28 binding at representative gene loci; the data shown are a representative result for more than three independent experiments. **e** WT or *Trim28*^*−/−*^ naive CD4^+^ T cells were cultured at Th17 condition (TGF-β plus IL-6) for 3 days, and then prepared for ChIP-qPCR assay performed by anti-H3K4Me3 antibody or MeDIP assay performed by 5hmc antibody. All error bars represent SDs. The experiments were repeated twice with consistent results

To investigate the direct targets of TRIM28 in Th17 cells, we performed chromatin immunoprecipitation coupled with high-throughput sequencing (ChIP-seq) assay. Compared with the mouse genome, an obvious enrichment of TRIM28-binding sites was found in the gene promoters (3 kb upstream and downstream of the transcription start site (TSS), 11% of TRIM28 binding sites vs. 2% of the mouse genome), exons (3 vs. 2%), introns (46 vs. 20%) and downstream regions (3 kb downstream of transcription ending sites, 2%), while 38% were localized in the intergenic regions (76% in the mouse genome) (Fig. 4a, Supplementary Fig. 4a).

Epigenetic mechanisms regulate gene expression by controlling chromatin structures and DNA accessibility. Global mapping of histone modifications and DNA methylation/demethylation have clearly demonstrated their involvement in regulation of signature gene expression in T helper cells^17,18^. As a well-known co-repressor, TRIM28 has been reported to facilitate repressive histone mark H3K9Me3 establishment and DNA methyltransferases binding^26,31^. To our surprise, comparison of TRIM28 ChIP-seq results with previous published Th17 ChIP-seq data^17,18^ revealed that TRIM28 bindings were largely co-localized with active epigenetic marks H3K4Me3 and DNA hydroxyl-methylation (5hmc), but not repressive marks H3K27Me3 and DNA methylation (5mc) (Fig. 4b, c), suggesting that TRIM28 may also function as an activator instead of being a co-repressor.

Next, we closely examined the genes bound by TRIM28. The *Il17-Il17f* locus was highly enriched with TRIM28 binding peaks that were co-localized with the H3K4Me3 and 5hmc modifications (Fig. 4c, d), consistent with the positive role of TRIM28 in regulating *Il17* and *Il17f* expression in Th17 cells. However, although IL17^+^GM-CSF^+^ T cells were reduced after TRIM28 deletion in vivo, there was no binding peak at the *Csf2* gene locus (Supplementary Fig. 4b), suggesting an indirect regulation of GM-CSF expression by TRIM28. In addition, TRIM28 peaks were identified in the promoter and/or intron regions of the *Il21*, *Il23r*, *Rorc*, *Rorα*, *Batf*, and *Irf4* genes, which largely correlated with active histone modifications (Supplementary Fig. 4b), indicating TRIM28 may broadly regulate Th17 transcription program, though not all of these genes were downregulated in expression in the absence of TRIM28 (Supplementary Tables 1, 2).

We then investigated the effect of TRIM28 deficiency on chromatin modifications. As expected, *Trim28* deficiency resulted in reduction of active epigenetic marks in the *Il17-Il17f* locus, including H3K4Me3 and 5hmC (Fig. 4e). In line with this, a dramatic decrease of RNA polymerase II recruitment was observed at the *Il17-Il17f* locus in KO Th17 cells (Supplementary Fig. 4c). Therefore, the co-localization of TRIM28 with active epigenetic marks and decrease of active epigenetic marks as a result of its ablation suggest that TRIM28 functions as an epigenetic activator in Th17 cell differentiation.

### TRIM28 regulates super-enhancers during Th17 differentiation

**Fig. 5:**
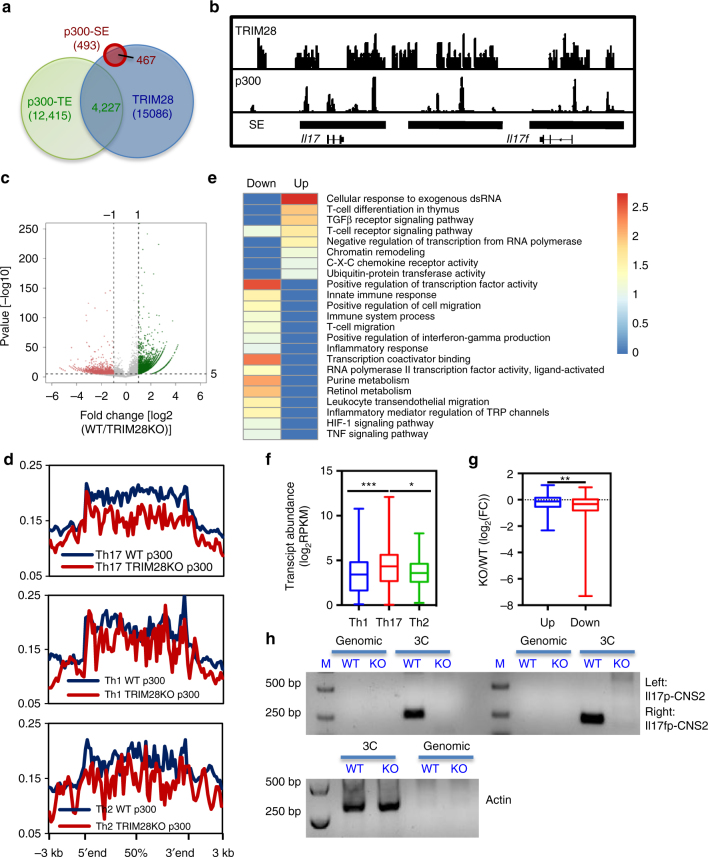
TRIM28-deficiency impaired epigenetic activation in Th17 cells. TRIM28 ChIP-seq was performed as in Fig. 4. **a** Overlap of TRIM28 binding peaks with SE and TE regions in Th17 cells. **b** IGV browser view of TRIM28, p300 binding peaks and SE regions at the *Il17-Il17f* gene locus (right). **c** Volcano plot showing p300 binding peak change in WT and *Trim28*^−^^*/*^^−^ Th17 cells cultured for 3 days (TGF-β+IL-6). **d** Overlay of p300 binding peaks in WT and TRIM28KO Th17 cells over Th1-, Th2 and Th17-specific SEs with decreased p300 intensity. **e** Heatmap of pathway analysis in genes associated with Th17-SEs that had decreased (left) or increased (right) p300 recruitment in KO cells. **f** Transcriptional comparison of T helper-specific SE-associated genes analyzed from WT Th17 RNA-seq data (*p* values, Mann−Whitney test). **g** Fold change of Th17-SE-related genes with increased (up) or decreased (down) p300 binding at the SE regions (*p* values, Mann-Whitney test). **h** 3C-PCR experiments were performed in WT and TRIM28KO Th17 cells cultured as in **c** and the PCR products were acquired by nest PCR. M is short for marker, and the whole DNA gels are shown in Supplementary Fig. 8. *p < 0.05, **p < 0.01, ***p < 0.005. All error bars represent SDs. The experiments were repeat twice with consistent results

Cell type-specific SEs have been associated with cell fate decision. In CD4^+^ T cells, SEs were reported to be enriched at genes encoding cytokines and their receptors, as measured by p300 loading intensity^20^. Interestingly, more than 40% of TRIM28 binding peaks in Th17 cells were marked by p300 bindings (Supplementary Fig. 5a). Since the expression of cytokines was preferentially defective in *Trim28*^*−/−*^ Th17 cells, we further examined whether TRIM28 regulates SE establishment. Based on the published method^20^ and p300 ChIP-seq data^32^, we first identified 493 SEs in Th17 cells (Fig. 5a), including the *Il17-Il17f* locus (Fig. 5b), then overlaid them with TRIM28 ChIP-seq data. Interestingly, the SEs were largely associated with TRIM28 bindings, whereas TE, characterized by lower p300 intensity, had much less correlation with TRIM28 bindings (Fig. 5a).

To understand the potential role of TRIM28 in SE establishment during Th17 differentiation, we assessed the enhancer activation after *Trim28* deletion. Compared with WT Th17 cells, a dramatic decrease of p300 recruitment was observed at the *Il17-Il17f* locus in *Trim28*^*−/*^^−^ Th17 cells (Supplementary Fig. 5b). In addition, both the H3K27Ac and H3K4Me1 modifications, markers for active or poised enhancers, respectively, were decreased at the *Il17-Il17f* locus as well (Supplementary Fig. 5b). To further analyze genome-wide p300 binding, we performed ChIP-seq experiments in WT and *Trim28*^*−/−*^ Th17 cells and found *Trim28* deficiency led to a global loss of p300 recruitment (Fig. 5c). As reported, each T helper subset bears a unique SE architecture^20^, so we further compared our ChIP-seq data with the lineage-specific SEs. Interestingly, the reduction of p300 binding in the absence of TRIM28 occurred preferentially at Th17 signature SE regions (Fig. 5d), within which the associated genes were largely Th17 signature genes, as indicated by pathway analysis (Fig. 5e), with obviously higher transcriptional activity (Fig. 5f). In contrast, *Trim28* deficiency had much less pronounced effect on p300 intensity at Th1 and Th2 signature SE regions in Th17 cells (Fig. 5d). In addition, we noticed that *Trim28* deficiency resulted in a more pronounced reduction in the expression of Th17-SE genes with decreased p300 bindings (Fig. 5g). The histone marker H3K27Ac has also been utilized to characterize SEs in many studies. Thus, we compared H3K27Ac modifications in WT and TRIM28KO Th17 cells by ChIP-seq. Similar to the measurement with p300, loss of TRIM28 in Th17 cells resulted in a dramatic decrease of H3K27Ac intensity and a preferential H3K27Ac reduction at Th17 SEs that contain many Th17 signature genes or positive regulators of transcription (Supplementary Fig. 5c, d). Therefore, the above data indicate a positive role of TRIM28 in p300 recruitment and activation of Th17-specific SEs.

Interestingly, loss of TRIM28 globally decreased permissive epigenetic markers at the *Il17-Il17f* locus, including both the promoter region and the nearby conserved noncoding sequences like CNS2. Our group has reported the critical role of CNS2 in regulating chromosome-looping and epigenetic activation at the *Il17-Il17f* gene locus, through interacting with RORγt^33^. To determine whether TRIM28 also regulates the long-range chromosomal associations at the *Il17-Il17f* locus, the chromosome conformation capture(3C) assay was performed in WT and *Trim28*^*−/−*^ Th17 cells as previously reported^33^. Consistent with its role in regulating the *Il17* and *Il17f* gene expression, the physical interactions between CNS2 with the *Il17* or *Il17f* gene promoters were dramatically reduced in the absence of *Trim28* (Fig. 5h). Taken together, our data suggest an important function of TRIM28 in regulating the chromatin accessibility and shaping the 3D-structure of the *Il17-17f* locus.

### TRIM28 recruitment is required for RORγt function

**Fig. 6:**
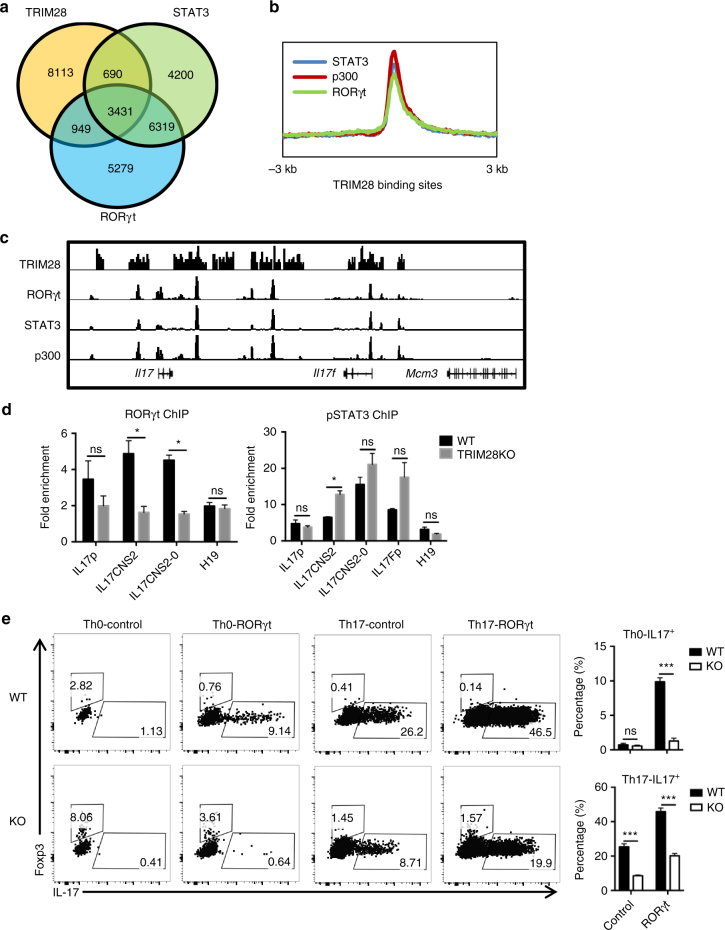
TRIM28 functions as a cofactor for RORγt and STAT3. **a** Overlay of TRIM28 binding peaks with that of RORγt and STAT3 in Th17 cells. **b** Genome-wide distributions of binding peaks of RORγt, STAT3, and p300 at TRIM28 binding sites. **c** IGV browser view of RORγt, STAT3, and TRIM28 binding peaks at the *Il17-Il17f* gene locus. **d** Binding of RORγt and STAT3 to the *Il17-Il17f* gene locus in WT and *Trim28*^*−/*^^−^ Th17 cells cultured at TGF-β plus IL-6 condition. **e** Overexpression of RORγt in WT or TRIM28 KO CD4^+^ T cells polarized under neutral (Th0) or Th17 (TGF-β plus IL-6) conditions. **d**, **e** The experiments were repeated 2–3 times with consistent results, and Student’s *t *test was used for the statistical test (ns = not significant; *p < 0.05, **p < 0.01, ***p < 0.005)

To understand the relationship of TRIM28 with Th17-related transcription factors, we compared the TRIM28 ChIP-seq result with previously published ChIP-seq data of transcription factors like IRF4, BATF, c-Maf, RORγt, and STAT3^32^. Interestingly, TRIM28 shared extensive amounts of common binding sites with Th17-related transcription factors, especially RORγt and STAT3 (Fig. 6a, b), where p300 were often occupied (Fig. 6a–c). Furthermore, these co-occupancy was even more obvious in Th17 signature genes selected from our microarray data (Supplementary Fig. 6a), suggesting that TRIM28, STAT3, and RORγt may share similar functions. In line with this, loss of either STAT3 or RORγt resulted in a dramatic reduction of active histone and DNA markers in Th17 cells (Supplementary Fig. 6b), similar to *Trim28*-deficient cells (Fig. 4e, Supplementary Fig. 5b).

The co-localization of TRIM28 with transcription factors prompted us to investigate the consequence of their recruitments. Using the *Il17-Il17f* locus as a model, we conducted ChIP with anti-pSTAT3 and anti-RORγt antibodies in either WT or *Trim28*^*−/*^^−^ Th17 cells. pSTAT3 binding at the *Il17-Il17f* locus was not affected, whereas RORγt recruitment was greatly compromised in the KO cells (Fig. 6d), suggesting TRIM28 acts downstream of STAT3 but upstream of RORγt in Th17 cells. RORγt is the lineage-specific transcription factor that drives *Il17* expression and Th17 differentiation even under unfavorable conditions^15^. However, RORγt overexpression in *Trim28*^*−/*^^−^ cells barely induced IL-17 expression in Th0 condition and only partially rescued the Th17 differentiation defect under Th17 polarizing condition (Fig. 6e). Additional overexpression of RORα, another Th17-restricted transcription factor with synergistic effects with RORγt, did not further rescue the defect (Supplementary Fig. 6c). Therefore, STAT3-dependent TRIM28 recruitment is required for RORγt recruitment and function at the *Il17-Il17f* locus.

### Cytokine signaling regulates TRIM28 recruitment

**Fig. 7:**
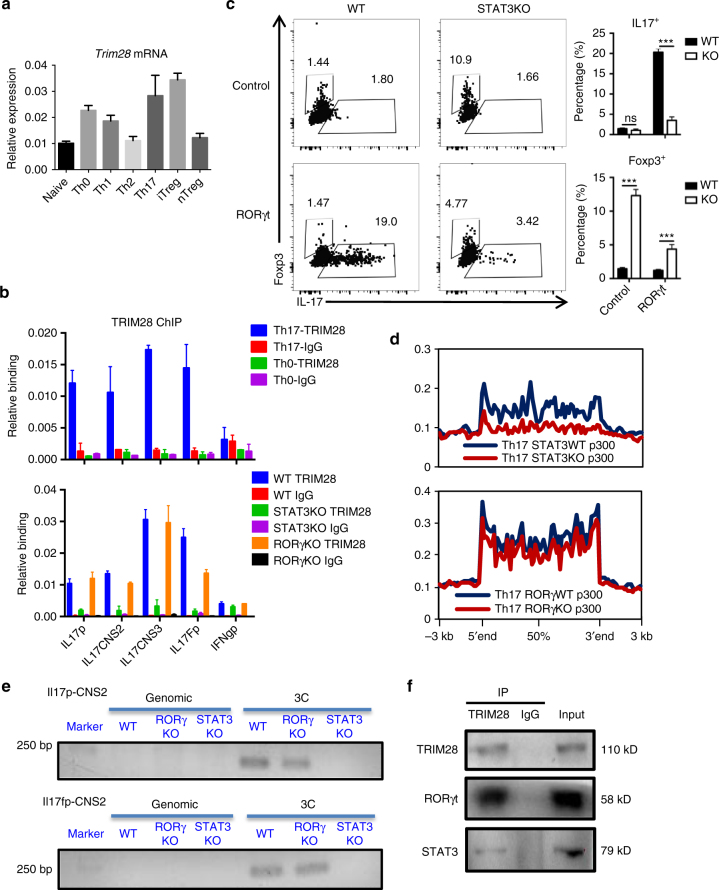
Cytokine signaling regulated TRIM28 recruitment. **a** Expression of TRIM28 in different T-cell subsets and naive CD4^+^ T cells. **b** TRIM28 ChIP-qPCR results in WT Th0, WT Th17, STAT3 KO Th17 or RORγt KO Th17 cells. **c** RORγt overexpression in WT or STAT3 KO CD4^+^ T cells polarized under neutral (Th0) condition. **d** Overlay of p300 binding peaks in WT/STAT3KO(Upper) or WT/RORγKO (lower) Th17 cells over Th17-specific SEs with decreased p300 intensity. **e** 3C-PCR experiments were performed in WT and TRIM28KO Th17 cells cultured for 3 days and the PCR products were acquired by nest PCR. M is short for marker, and the whole DNA gels are shown in Supplementary Fig. 8. **f** Immunoblot results of TRIM28 immunoprecipitated protein complexes as detected by RORγt and STAT3 antibodies in Th17 cells. The whole WB gels are shown in Supplementary Fig. 9. Those experiments were repeated twice with consistent results, and Student’s *t *test was used for the statistical test (ns = not significant; *p < 0.05, **p < 0.01, ***p < 0.005). All error bars represent SDs

To understand TRIM28 regulation in Th17 cells, we first examined its transcription level in different T-cell subsets. *Trim28* was constantly highly expressed in all of these cells, including naive CD4^+^ T cells, Th0 cells, Th1, Th2, Th17 cells, iTreg and nTreg cells (Fig. 7a), indicating TRIM28 is not regulated at the transcription level. In addition, we also tested the localization of TRIM28 protein during Th17 cell differentiation and found it was mainly localized in the nuclei in both naive and Th17 cells (Supplementary Fig. 7a). In accordance with these findings, the binding of TRIM28 to the *Il17-Il17f* locus was strictly restricted in Th17 cells, but not Th0 cells (Fig. 7b), indicating that Th17-specific TRIM28 recruitment is independent of TCR signaling. TGF-β-induced Smad activation and IL-6-induced STAT3 phosphorylation are important for initiating Th17 differentiation^34,35^. Interestingly, loss of STAT3, but not Smad2 or RORγt greatly impaired TRIM28 binding to the *Il17-Il17f* locus (Fig. 7b, Supplementary Fig. 7b). Moreover, knocking down the expression of the *Irf4* or *Batf* genes, which have been reported to initiate Th17 differentiation together with STAT3^32^, there was an obvious decrease of TRIM28 recruitment in those cells (Supplementary Fig. 7c, d). Taken together, the above findings indicate that TRIM28 recruitment in Th17 cells is dependent on STAT3 and its co-factors.

STAT3 has been shown to be necessary for inducing RORγt expression^14^. However, overexpression of RORγt could barely induce IL-17 expression in STAT3-deficient T cells (Fig. 7c, Supplementary Fig. 7e), similar to *Trim28*^*−/−*^ cells (Fig. 6e). Notably, genome-wide ChIP-seq analysis showed that STAT3-deficiency resulted in a systemically reduction of p300 recruitment, similar to TRIM28-deficincy, whereas RORγt deficiency only mildly affected p300 binding in Th17 cells (Fig. 7d, Supplementary Fig. 6d). In addition, p300 loading intensity in Th17-SEs and chromosome looping of the *Il17-Il17f* locus were also selectively reduced in *Stat3*^−/−^ but not *Rorc*^*−/−*^ Th17 cells (Fig. 7d, e, Supplementary Fig. 6d, 7f). Finally, we found that both RORγt and STAT3 could be readily co-immunoprecipitated by an antibody against TRIM28 in Th17 cells (Fig. 7f, whole gel in Supplementary Fig. 9).

Considering the sequential requirement of STAT3, TRIM28, and RORγt recruitment in Th17 cells and the inability of RORγt overexpression to rescue STAT3- or TRIM28-deficient T cells in IL-17 expression, these studies thus identified TRIM28 as a functional epigenetic mediator downstream of STAT3 in directing IL-17 expression and Th17 differentiation.

## Discussion

In this paper, we identified a crucial role of TRIM28 in regulating Th17 cell development both in vitro and in vivo. Despite TRIM28 does not regulate the expression of key Th17-related transcription factors, it controls RORγt recruitment to key Th17-cytokine gene loci. Mechanistically, IL-6 induces STAT3 activation and triggers subsequent TRIM28 recruitment, causing chromosome activation, looping of the *Il17-Il17f* locus and the binding of RORγt, then ultimately induces robust IL-17 production and Th17 differentiation.

STAT3, downstream of IL-6, IL-21 and IL-23 signaling, is required for RORγt expression in Th17 cells^14,16^. However, overexpression of RORγt could not rescue IL-17 expression in STAT3-deficient cells (Fig. 7c, Supplementary Fig. 7e), suggesting the activity of RORγt might be regulated by additional mechanisms, other than transcription. In this study, we first found that TRIM28, a large scaffold protein with multiple function domains, was required for Th17 differentiation in vitro and in vivo. *Trim28* deficiency led to impaired IL-17 production and greatly increased Foxp3 expression during Th17 differentiation, which closely mimics the phenotypes of STAT3-deficiency in previously studies^14,16^. Genome-wide analysis showed that TRIM28 shared a large cohort binding sites with many Th17-related transcription factors, particularly STAT3 and RORγt (Fig. 6a, b). ChIP studies demonstrated that the recruitment of TRIM28 to key Th17-speicific gene loci, including the *Il17-Il17f* locus, was dependent on STAT3, which in turn was required for RORγt loading onto its target genes in Th17 cells, but not vice versa (Figs. 6d, 7b). Our studies thus demonstrate that the recruitment of RORγt to its target sites is also tightly controlled by STAT3 that is bridged by TRIM28.

Super-enhancers, also called stretch-enhancers, are clusters of enhancers with unusual high occupancy of transcription factors and co-activators, and have recently been proposed as important regulatory regions controlling cell identity. Genes associated with SEs were found to be transcribed more abundantly than TEs and are highly cell-type specific^19^. The SEs have been characterized in Th1, Th2 and Th17 cells and found to be predominantly associated with genes of cytokines and cytokine receptors^20^. However, how cell-type-specific SEs are induced and maintained in T cells remained largely elusive. In our studies, we found that more than 90% of SE regions contained TRIM28 binding peaks in Th17 cells, while only 34% of TEs were occupied by TRIM28 (Fig. 5a). Interestingly, TRIM28 deficiency selectively affected p300 loading intensity in Th17 signature SEs, as well as the key SE-related epigenetic markers H3K27Ac (Fig. 5d, Supplementary Fig. 5b, c). In addition, these TRIM28-regulated Th17-SEs (whose p300 was reduced upon *Trim28* deletion) encode many genes involved in various immune functions (Fig. 5e) and were more affected by *Trim28* deficiency than those encoded by TRIM28-independent Th17-SEs (whose p300 binding was not decreased upon *Trim28* deletion) (Fig. 5g). These findings were also confirmed by genome-wide comparing another well-established SE marker-H3K27Ac in TRIM28-deficient Th17 cells (Supplementary Fig. 5c, d), therefore pointing a prominent role of TRIM28 in regulating super enhancer activities in Th17 cells.

TRIM28 was largely identified as a transcriptional co-repressor, by recruiting repressive epigenetic complexes and inducing chromatin condensation, or through its sumoylation and ubiquitin E3 activity^36^. However, comparison of ChIP-seq data in Th17 cells revealed highly correlated binding of TRIM28 with active epigenetic markers such as H3K4Me3 and 5hmC, as well as p300 binding, particularly in Th17-SEs. Genetic ablation of TRIM28 resulted in a global reduction of p300 recruitment and SE activity, as well as several key active histone and DNA markers H3K4me3, H3K4me1, H3K27Ac and 5hmC (Figs. 4e, 5c–g, Supplementary Fig. 5b). In total, TRIM28 deficiency affected 977 genes expression in Th17 cells (FDR < 0.05, fold change > 2), in which 652 (67%) were reduced in TRIM28-deficient Th17 cells and 48% of them contain TRIM28 binding peaks, whereas only 325 (33%) genes had increased expression with less than 25% having TRIM28 binding peaks (Supplemental Tables 1, 2). These data together suggest that TRIM28 largely functions as an epigenetic activator in Th17 cells. Transcriptome analysis also found that TRIM28-deficiency led to impaired transcription of many Th17 signature genes, without any master repressors identified (Fig. 2b–e). Therefore, our study indicates that TRIM28 mainly functions as a co-activator in Th17 development. In addition, ChIP and co-IP studies demonstrate that TRIM28 formed a complex with the key transcription factors STAT3 and RORγt in Th17 cells (Figs. 6a−d, 7b, f), suggesting that the co-activator or co-repressor function of TRIM28 might be fine-tuned by context-dependent interacting partners, or through distinct posttranslational modifications as previously reported^37–39^.

In contrast to our findings, a previous study using the proximal Lckcre-mediated deletion strategy suggested TRIM28 as a negative regulator of IL-17 and autoimmune syndrome^27^. However, this study did not examine the direct effect of TRIM28 on Th17 differentiation, and their in vivo findings could be largely attributed to disrupted Treg functions. Through using the in vitro culture system and T-cell transfer colitis, our study first identified a T-cell intrinsic effect of TRIM28 in directing Th17 differentiation. With the help of our recently generated Il17fcre mice^17^, we found that selective ablation of TRIM28 in maturing Th17 cells did not cause increased Foxp3 expression but yet still resulted in impaired IL-17 expression in vitro and in vivo, and caused resistance to EAE, thus establishing a Th17-intrinsic effect of TRIM28 in regulating Th17 development (Fig. 3). Besides, proliferation was reported to be reduced in the *Trim28*^*fl/fl*^Lckcre CD4^+^ T cells. We also tested this in our *Trim28*^*fl/fl*^CD4cre CD4^+^ T cells under different TCR stimulations in vitro. Although there was a mild defect of proliferation ability in the KO cells stimulated with low TCR agonist, as indicated by CFSE dilution (88% CFSE^low^ in WT vs. 66% in KO), we did not observe obvious defects under high-dose TCR stimulation (98% CFSE^low^ in WT vs. 93% in KO) (Supplementary Fig. 3e). Importantly, we observed strong Th17 differentiation defects in KO cells under all TCR stimulation tested; thus there might not be a proliferation defect in KO cells to contribute to its Th17 differentiation defect. In addition, *Trim28*^*fl/fl*^Lckcre CD4^+^ T cells had decreased IL-2 and increased TGF-β expression when stimulated in vitro, which were not or slightly affected in our experiments using *Trim28*^*fl/fl*^CD4cre Th17 cells (Fig. 2b, Supplementary Fig. 3c). This inconsistency is possibly due to different environmental and cellular context. In the previous paper, IL-2 production was measured by knockdown in cell line or total CD4^+^ T cells sorted from KO mice possibly with inflammation, and both cytokines were compared only under neutral conditions in vitro. In addition, TGF-β is widely secreted by various kinds of cells and its mRNA does not always correlate with protein level^40^, thus the elevated TGF-β mRNA expression in *Trim28*^*fl/fl*^Lckcre CD4^+^ T cells in vitro may not cause obvious biological consequence in vivo.

Interestingly, although increased FOXP3 expression was observed in the *Trim28*^*fl/fl*^CD4cre during Th17 cell differentiation, suppressing FOXP3 upregulation either by increasing IL-6 concentration or adding IL-2 blocking antibody still could not restore the IL-17 production in KO cells (Supplementary Fig. 3a, d), suggesting the Th17 differentiation defect are cell intrinsic and independent of FOXP3 suppression. Consistent with our findings, dysfunctional Treg cells were observed after TRIM28 deletion, despite its increase of percentages^27^. Meanwhile, Rudra reported in human Treg cells, TRIM28 could complex with FOXP3 through the bridging of FIK, and disruption of the TRIM28−FIK−FOXP3 interaction abrogated the suppressive function of Treg cells^41^. In addition, we considered the possibility of direct regulation of *Foxp3* expression by TRIM28. However, although we found a single TRIM28 binding peak at the last exon of the *Foxp3* gene in Th17 cells in our ChIP-seq study, it is not located in the promoter or *Foxp3* gene regulatory regions CNS1-3, or co-localized with active epigenetic modifications H3K4Me3 or 5hmc (Supplementary Fig. 4b), suggesting that TRIM28 might regulate *Foxp3* gene expression indirectly.

In summary, we identified TRIM28 as a critical positive regulator of Th17 development, in which TRIM28 serves as an epigenetic activator downstream of STAT3 in response to cytokine signaling, and directs Th17 differentiation by regulating chromosomal activation and 3D-looping of key effector cytokine genes, and highlights epigenetic intervention as possible therapeutic approaches in treatment of Th17-related autoimmune diseases.

## Methods

### Animals

*Trim28*^*fl/fl*^ mice were described before^28^ and bred with CD4cre mice^42^ and Il17fcre *mice*^17^ for making different conditional knockout mice. *Stat3*^*fl/fl*^^43^, *Rorc*^*fl/fl*^^44^ and *Smad2*^*fl/fl*^^45^ mice were all crossed with CD4cre mice to generate conditional knockout mice. *Rag1*^*−/−*^ mice were obtained from the Jackson Lab (Stock number: 002216). All mice are on B6 background, fed in the SPF animal facility at Tsinghua University and killed by CO_2_. All the animal experiments were performed with the use of protocols approved by the Institutional Animal Care and Use Committee of Tsinghua University. All the animal studies were done without blinding or randomization, and sample sizes were chosen based on experience.

### Naive T-cell isolation and in vitro differentiation

Naive T cells were isolated by sorting CD4^+^CD25^−^CD62L^hi^CD44^low^ cells from spleens and lymph nodes (seen gating strategy in Supplementary Fig. 10a), and stimulated with the plate-bound anti-CD3 (clone 17A2; BioXCell, 5 μg ml^−1^) and anti-CD28 (clone 37.51; BioXCell, 5 μg ml^−1^) under different cytokine cocktails. The T cells were cultured with anti-IFN-γ (clone XMG1.2; BioXCell, 10 μg ml^−1^) and anti-IL-4 (clone 11B11; BioXCell, 10 μg ml^−1^) for Th0 differentiation, mIL-12 (Peprotech; catalog 210-12, 10 ng ml^−1^) and anti-IL-4 (10 μg ml^−1^) for Th1 differentiation, mIL-4 (Peprotech; catalog 214-14, 10 ng ml^−1^) and anti-IFN-γ (10 μg ml^−1^) for Th2 cell differentiation, hTGF-β1 (R&D Systems; catalog 240-B-010, 2 ng ml^−1^) with IL-2 (Peprotech; catalog 212-12, 10 U ml^−1^) for iTreg differentiation. The Th17 differentiation was performed using hTGF-β1 (0.5 ng ml^−1^) and IL-6 (Peprotech; catalog 216-16, 20 ng ml^−1^), with or without IL-1β (Peprotech; catalog 211-11B, 10 ng ml^−1^) and IL-23 (R&D Systems; catalog 1887-ML, 50 ng ml^−1^) as indicated.

### EAE and colitis

The EAE disease was induced and analyzed as previously described^33^. Briefly, the mice were immunized (s.c injection on both dorsal sides) with 150 μg MOG_35–55_ peptide in complete Freund’s adjuvant (Sigma) twice on day 0 and day 7, and 500 ng pertussis toxin (PTX, dissolved in 0.1 ml of PBS) was injected intraperitoneally the next day following immunization. The mice were monitored daily, and the disease severity was scored as follows: 0, no clinical sign of disease; 1, loss of tail tonicity; 2, wobbly gait; 3, complete hind limb paralysis; 4, complete hind and fore limb paralysis; 5, moribund or dead.

For colitis induction, the CD4^+^CD25^−^CD62L^hi^CD45RB^hi^ T cells were sorted by flow cytometry (seen gating strategy in Supplementary Fig. 10a) and transferred intravenously into *Rag1*^*−/−*^ mice (0.4×10^6^ cells per mouse) and the weight loss was monitored weekly. The mice were killed 2–3 months later and leukocytes from the lamina propria (LPL), lymph nodes and spleen were isolated and re-stimulated by PMA, ionomycin and Golgiplug for FACS analysis.

### RNA extraction and RNA-seq

WT or *Trim28*^*−/−*^ Th17 cells cultured with TGF-β and IL-6 were re-stimulated with anti-CD3 after 3 days. Total RNA was extracted by TRIZOL (Life Technologies) according to the manufacturer’s instructions and sent to BerryGenomics for deep sequencing. The sequencing reads were mapped to the *Mus musculus* genome (version mm10) using SOAP2 without quality problems. RPKM (Reads Per Kilobases per Million reads) was used to calculated genes profiling for WT or *Trim28*^*−/−*^ Th17 cells and differentially expressing genes were obtained by following the previous published method with at least twofold change and FDR lower than 0.05.

### ChIP-seq and data analysis

ChIP experiment was done following instructions of Active Motif’s ChIP assay kit (53035) with slight modifications, in which ten cycles of sonication (Bioruptor) were added after enzyme digestion, and dynabeads protein G (Life Technologies) were used for immunoprecipitations. The antibodies used for ChIP including: anti-TRIM28 (Active Motif, 61173), rabbit IgG (Abcam, ab37415), anti-pSTAT3 (CST, 9134), anti-RORγt (Abcam, ab78007), anti-p300 (Santa Cruz, sc-585), anti-phospho-PolII(Ser5)(CST, 13523), anti-H3K4me3 (Abcam, ab8580), anti-H3K27me3 (Millipore, 07-499), anti-H3K4me1 (homemade by Dr Wei Xie’s lab at Tsinghua University) and anti-H3K27ac (Active Motif, 39133). The precipitated DNA was quantified by real-time PCR or amplified with the NEXTflex^TM^ ChIP-Seq DNA Sequencing Kit (Bioo Scientific, 5143) for deep sequencing by companies (TRIM28 and H3K27Ac ChIP-seq were performed by BGI and p300 ChIP-seq by BioNova). Primers used for ChIP-qPCR are listed in Supplementary Table 3.

To analyze the sequencing data, the sequencing reads were mapped to the *Mus musculus* genome (version mm10) using Bowtie (version 1.0) with no more than two mismatches. After PCR duplicates removal by samtools, SICER (version 1.1) algorithm was used for peak calling, compared with each input control with FDR less than 1E-5. To accurately delineate the SE regions based on mm10, we followed the same approach proposed before^20^, and SE regions were obtained by the ROSE software with the following setting: -s 12500 –t 2500. To identify differential enriched regions, SICER-df was used with FDR = 1E-5. ChIP-seq data of RORγt, STAT3, p300, IRF4, BATF, H3K4me3 and H3K27me3 in Th17 cells were described before^18,32^.

### MeDIP

The MeDIP experiment was performed as previously described^17^. Briefly, the genomic DNA was purified and sonicated to size of 200–500 bp. DNA fragments were denatured and incubated with antibody against 5mC (Eurogentec), 5hmC (Active Motif), or control IgG(Abcam, ab37415) at 4 °C overnight. The precipitated DNA fragments were analyzed by qPCR. Primers used for MeDIP-qPCR are listed in Supplementary Table 3. MeDIP seq data were described before^17^.

### Chromosome conformation capture

The experiment was done following the published protocol^46^, with slight modifications, in which PstI and NsiI were used for restriction sites generation^33^ and nested PCR was used for detecting the ligation bands. Undigested Th17 genomic DNA and the *actin* locus^47^ were used for negative or positive controls for PCR reactions, respectively. *Il17p-CNS2* interaction was detected by first using primers *CNS2*-5′end-1 and *IL17p*-3′end-1, following by PCR with *CNS2*-5′end-2 and *IL17p*-3′end-2. Similarly, *Il17fp-CNS2* interaction was detected by two pairs of primers: *CNS2*-5′end-1 and *IL17fp*-3′end-1 first, then *CNS2*-5′end-2 and *IL17fp*-3′end-2. PCR primers are listed in Supplementary Table 4 and whole DNA gels are shown in Supplementary Fig. 8.

### Co-immunoprecipitation and immunoblot analysis

Th17 cells (2×10^7^) cultured under TGF-β and IL-6 for 3 days were resuspended in 1 ml lysis buffer (25 mM Τris, pH 7.4, 150 mM NaCl, 1 mM EDTA, 1% NP-40, 5% glycerol plus proteinase inhibitors) and incubated on ice for 30 min. Cells were sonicated for 5 cycles, pelleted by centrifugation, and 10 μl anti-TRIM28 antibody (CST, catalog 4123) or control IgG antibody was added to and incubated with the supernatant at 4 °C overnight. The next day, the immune complex was captured by Dynabeads protein G, washed thoroughly by lysis buffer for four times and eluted by adding 1× SDS sample loading buffer at 95  °C for SDS-PAGE fractionation and immunoblot analysis. The following antibodies were used for immunoblotting: anti-TRIM28 antibody (CST, catalog 4124), anti-RORγt (ebioscience, 14-6988-82), anti-STAT3 (CST, 12640).

### Statistical analysis

The statistical significance was determined by Student’s *t* test (two-tailed, *p < 0.05; **p < 0.01; ***p < 0.005), unless otherwise specified. All error bars shown in this article represent SDs. The variance between groups in all the experiments were similar.

### Data availability

All the RNA-seq and ChIP-seq data have been deposited in the GEO database under the accession codes: GSE98427 and GSE108598. All data generated or analyzed during this study are included in this published article and its supplementary information files.

## Electronic supplementary material

Supplementary Information

Peer Review File
