# Supplementary material for: Epigenetic activation during T helper 17 cell differentiation is mediated by Tripartite motif containing 28
Source: Nat Commun. 2018 Apr 12;9:1424. doi: 10.1038/s41467-018-03852-2 (PMC5897371; doi:10.1038/s41467-018-03852-2)
Supplement: Supplementary file 1 — Supplementary Information [file 41467_2018_3852_MOESM1_ESM.pdf]

Epigenetic activation during T helper 17 cell differentiation is mediated by Tripartite motif containing 28

Jiang et al.

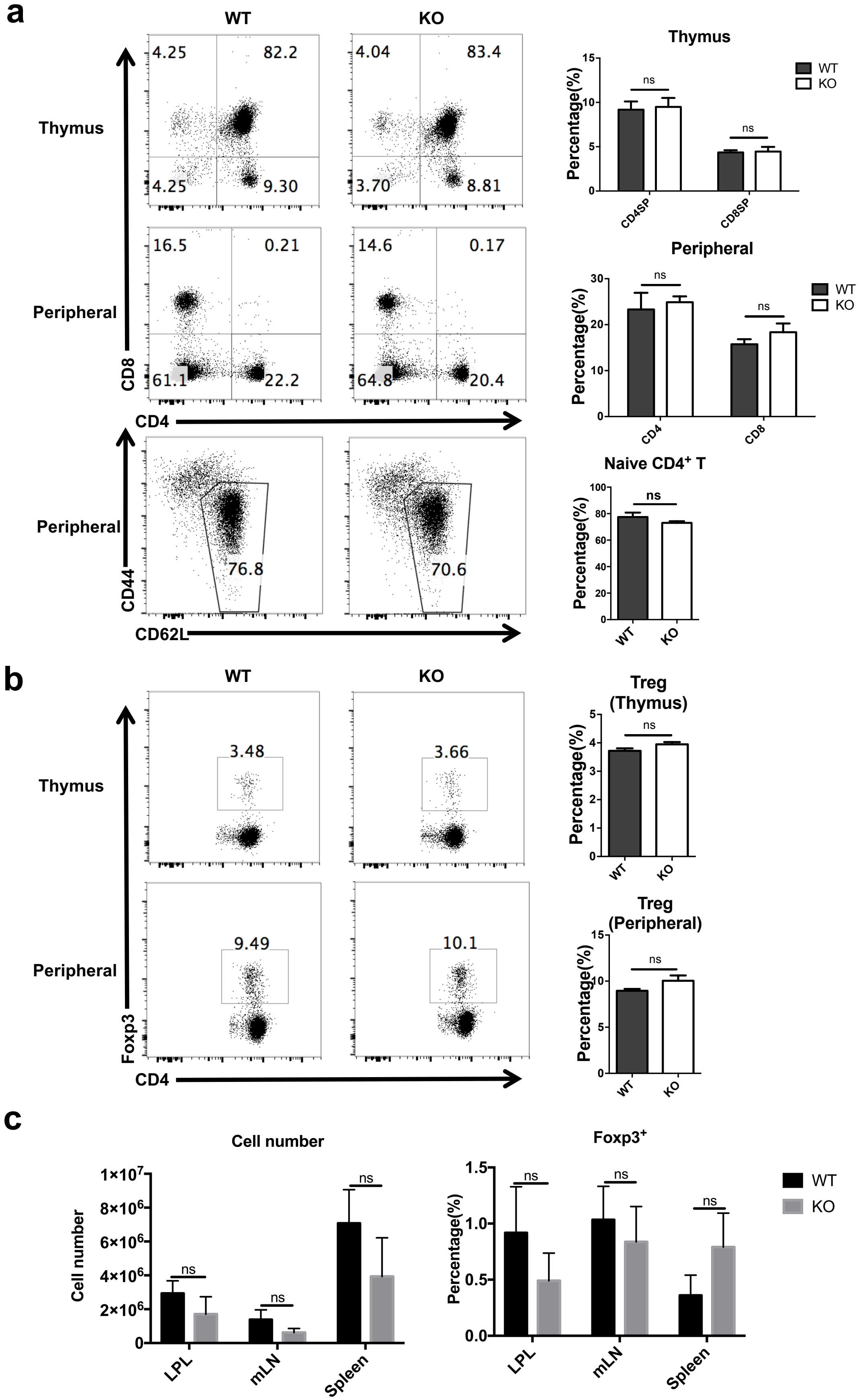

Supplementary figure 1. T cell homeostasis in *Trim28*$^{-/-}$ mice.

(a) Up: CD4+ and CD8+ T cells in the spleen, lymph nodes and thymus of WT (n=3) and *Trim28-/-* mice (n=3) (~6-7 weeks old); Down: naive CD4+ T cells in the peripheral lymph nodes of WT (n=3) and *Trim28-/-* mice (n=3) (~21 weeks old mice); **(b)** nTreg cells in the thymus and peripheral in WT (n=3) and *Trim28-/-* mice (n=3) (~6-7 weeks old); **(c)** Statistic data of total infilrated CD4+ T cell numbers and Treg cells in the lamina propria (LPL) of large intestine, mesenteric lymph nodes (mLN) and spleen isolated from colitis mice (WT: n=6, KO: n=4). The data are a representative for 2-3 independent experiments. The statistical significance was determined by Student's t test (ns, not significant; $p < 0.05$, *; $p < 0.01$, **; $p < 0.005$, ***), and all error bars represent SDs.

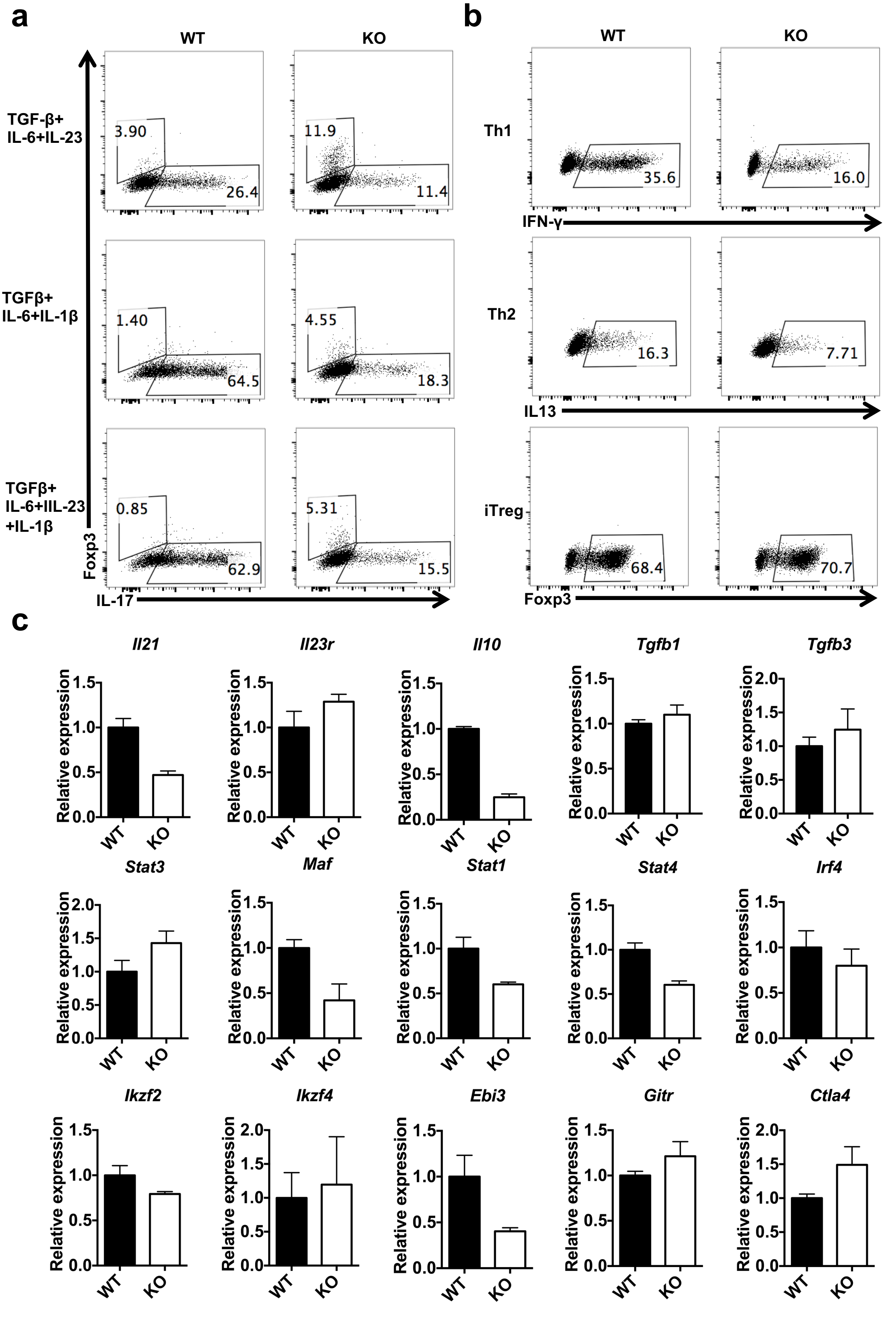

Supplementary figure 2. TRIM28-deficient T cells were defective in Th17 and Th1 differentiation. Naive CD4+ T cells were sorted from WT and *Trim28-/-* mice and then polarized into **(a)** Th17 cells under indicated conditions for 3 days, then re-stimulated for intracellular staining or **(c)** mRNA analysis(TGF-β+IL-6), and **(b)** Th1, Th2 and iTreg cells for 4-6 days in *in vitro* cultures, and analyzed by intracellular staining after re-stimulation. These experiments were repeated for 3 times with the consistent results. All error bars represent SDs.

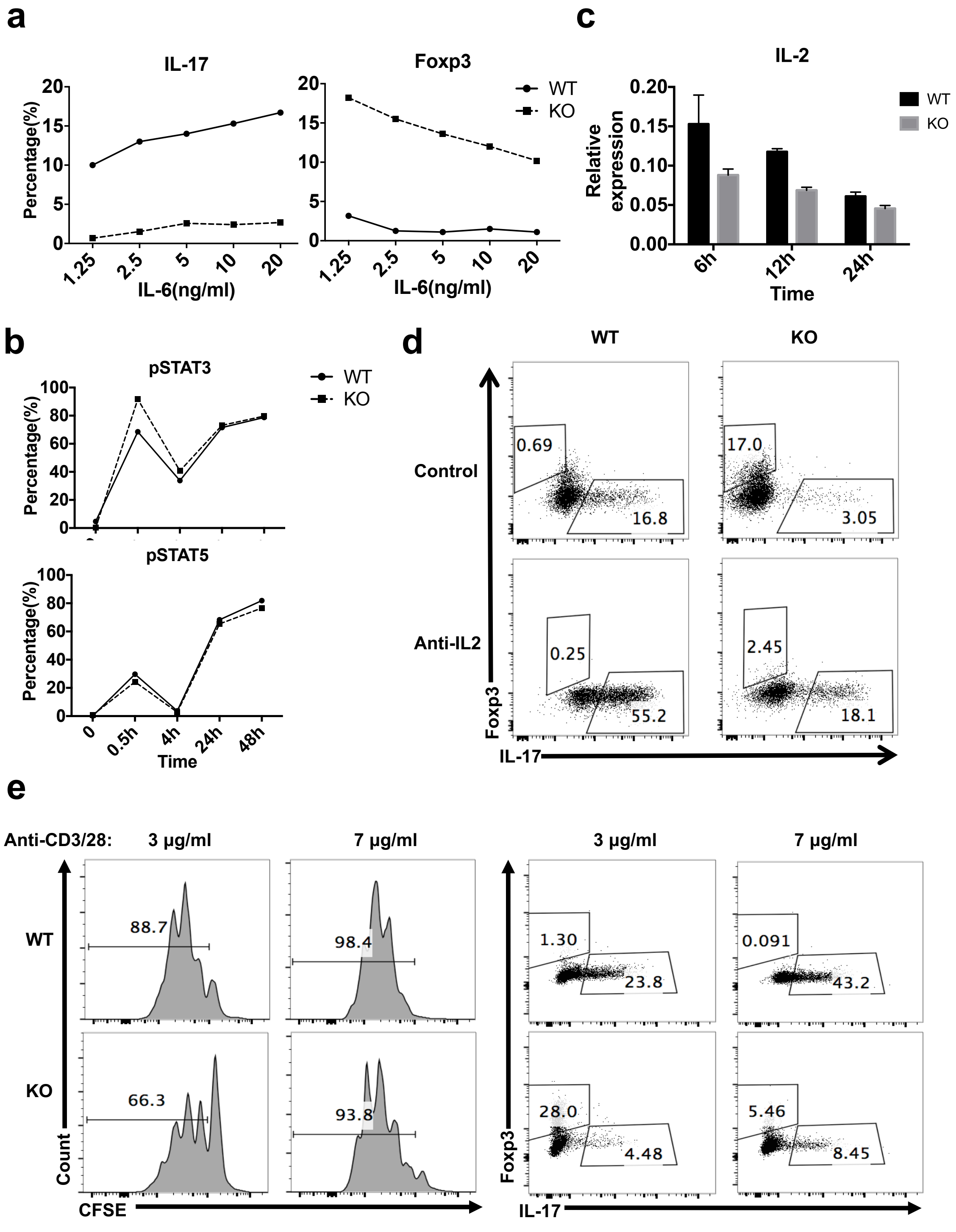

Supplementary figure 3. TRIM28 intrinsically regulates Th17 cell development.

(a-d) WT and *Trim28*$^{-/-}$ naive T cells were polarized into Th17 cells in the presence of TGF-β and IL-6. **(a)** Expression of IL-17 and Foxp3 as determined by intracellular staining under different IL-6 concentrations; **(b)** Intracellular staining of phosphorylated STAT3 and STAT5 in Th17 cells polarized at different time points; y-axis: percentage of phosphorylated STATs proteins, x-axis: time after T cell activation; **(c)** IL-2 mRNA expression at different time points as determined by real-time PCR; **(d)** Th17 differentiation were performed with or without IL-2 blocking antibody; **(e)** CFSE-labeled WT or *Trim28*$^{-/-}$ naive CD4$^+$ T cells were cultured with indicated concentrations of anti-CD3&CD28 stimulation in the presence of TGF-β and IL-6 for 3 days, and then harvested for CFSE detection (left) and intracellular staining (right). These experiments were repeated for twice with the consistent results. All error bars represent SDs.

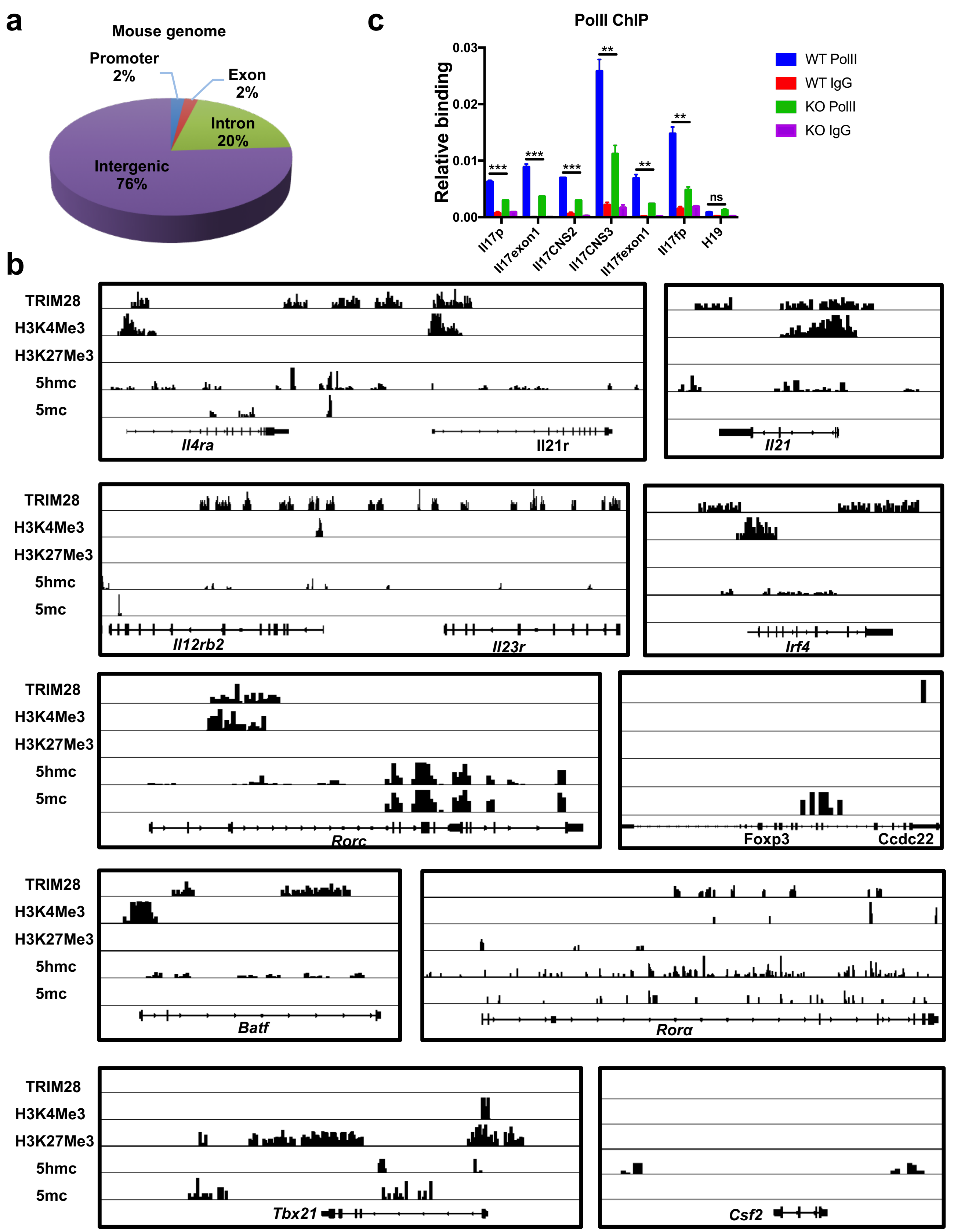

Supplementary figure 4. TRIM28 positively regulated epigenetic activation in Th17 cells.
(a) Distribution of genetic features across the whole mouse genome (mm10); **(b)** IGV browser view of histone markers (H3K4me3 & H3K27me3) and DNA markers (5hmc & 5mc) and TRIM28 binding peaks at the indicated gene loci in WT Th17 cells cultured *in vitro;* **(c)** WT or *Trim28*$^{-/-}$ naive CD4^{+} T cells were cultured at Th17 condition (TGF-β plus IL-6) for 3 days, and then harvested for ChIP-qPCR by anti-phospho-PolII antibody (Ser5). This experiment was repeated twice with consistent results. The statistical significance was determined by Student's t test (ns, not significant; p < 0.05, *; p < 0.01, **; p < 0.005, ***), and all error bars represent SDs.

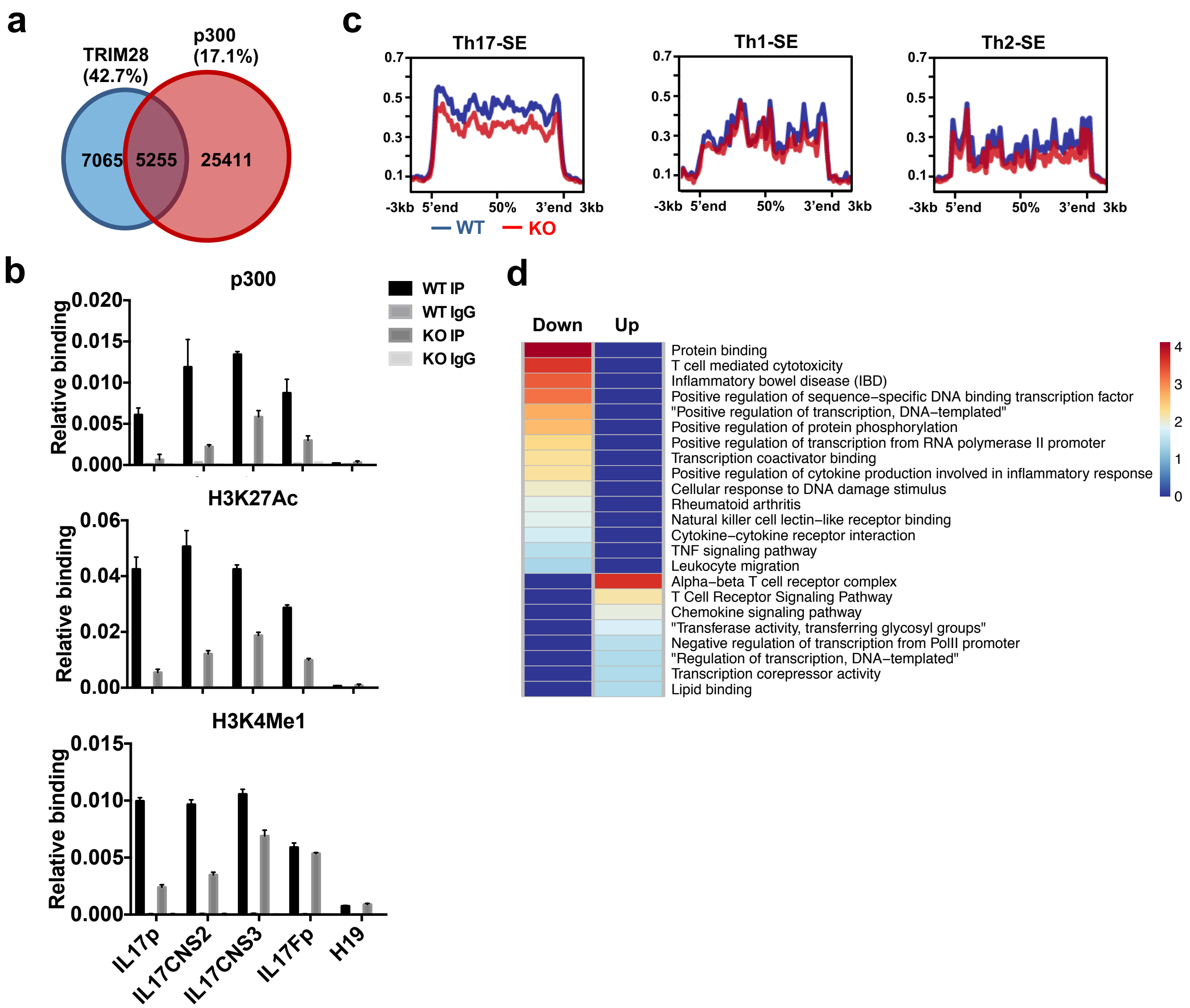

Supplementary figure 5. TRIM28 regulates super-enhancer establishment in Th17 cells.

(a) Overlap of TRIM28 and p300 binding peaks in Th17 cells; **(b-e)** WT or *Trim28*$^{-/-}$ naive CD4^{+} T cells were cultured at Th17 condition (TGF-β plus IL-6) for 3 days, and then prepared for ChIP-qPCR **(b)** or ChIP-seq experiments **(c-d)** performed with indicated antibodies. **(c)** Peak overlay of WT/TRIM28KO H3K27Ac marker over Th-sepcific SEs; **(d)** Heatmap of pathway analysis in genes associated with Th17-SEs that had decreased(left) or increased(right) H3K27Ac peaks. The qPCR were repeated 3 times with consistent results. All error bars represent SDs.

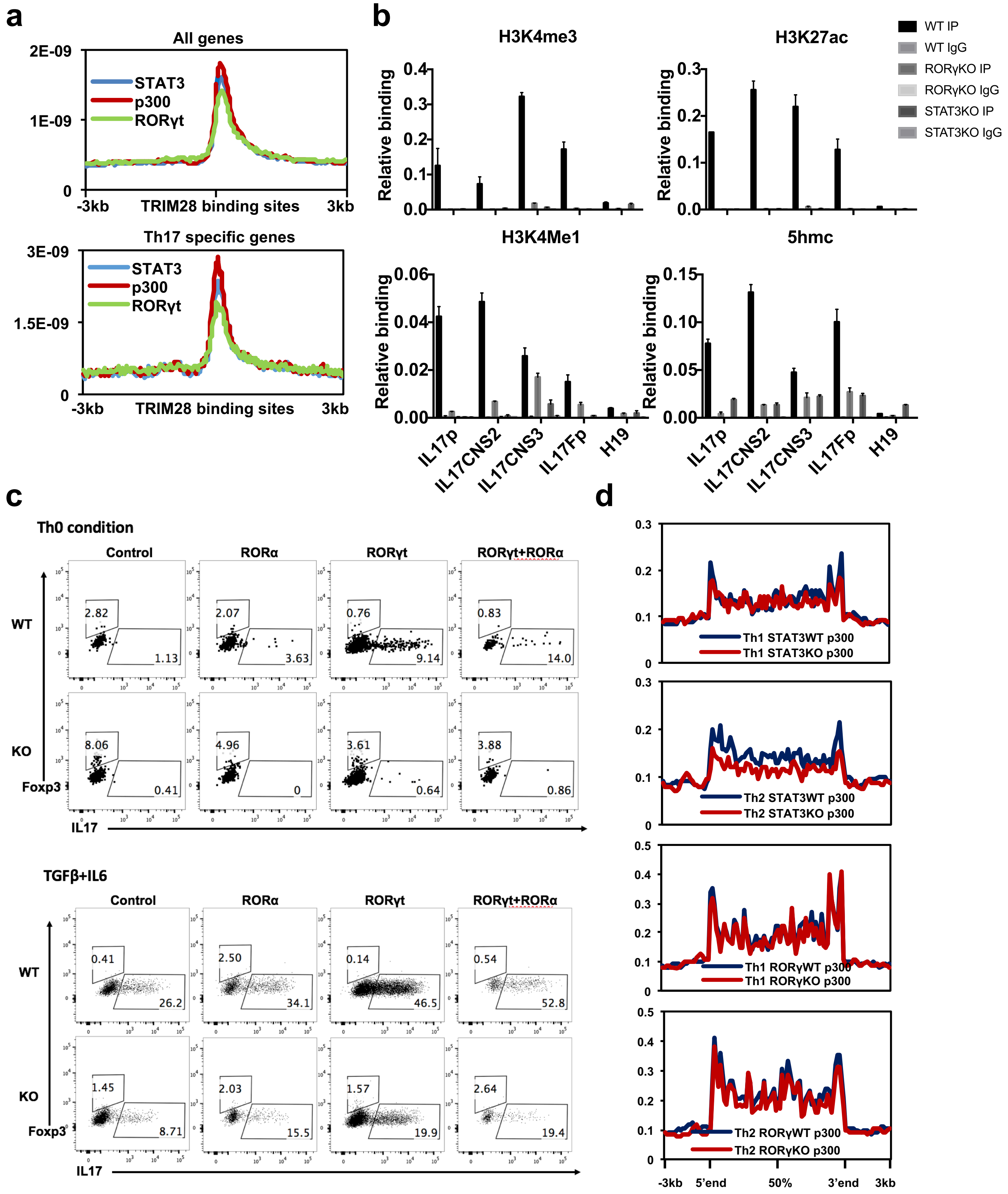

Supplementary figure 6. TRIM28 bound to STAT3 and RORγt occupied regions in Th17 cells.
(a) Overlay of TRIM28 binding peaks with that of STAT3, RORγt and p300 in Th17 cells in all genes(up) vs Th17-specific genes (down); **(b)** Effect of STAT3- or RORγ-deficiency on changes of histone and DNA markers in Th17 cells, the error bars represent SDs; **(c)** Overexpression of RORγt and RORα alone or together in WT and TRIM28 KO CD4⁺ T cells polarized under Th0 or Th17 (TGF-β plus IL-6) conditions; **(d)** Overlay of p300 binding peaks in STAT3 WT/KO cells and RORγ WT/KO cells at Th1-SE and Th2-SE regions. **(b-c)**, the experiments were repeated twice with consistent results.

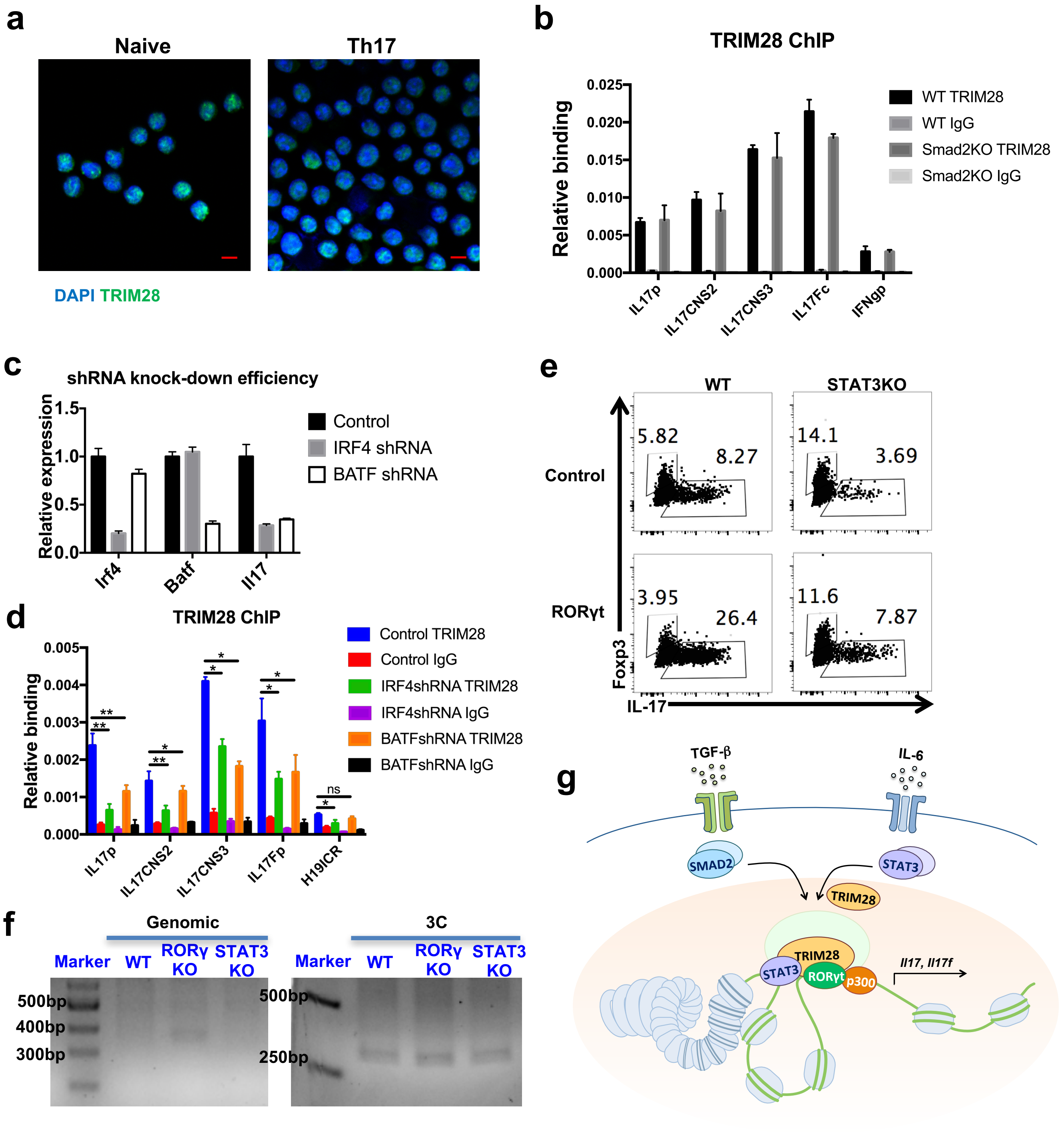

Supplementary figure 7. Functional cooperation between TRIM28, STAT3 and RORγt in Th17 cells.
(a) Naive CD4⁺ T cells and Th17 cells cultured for 3 days (TGF-β plus IL-6) were fixed for immunofluorescence staining (scale bars: 0.5 μm); **(b)** TRIM28 ChIP-qPCR results in WT and Smad2 KO Th17 cells; **(c)** Knock-down efficiency of IRF4 and BATF shRNAs; **(d)** TRIM28 ChIP-qPCR results in control and Irf4/Batf gene knock-down Th17 cells; **(e)** RORγt overexpression in WT and STAT3KO CD4⁺ T cells polarized under Th17 (TGF-β plus IL-6) condition; **(f)** 3C results of control actin locus in WT, STAT3KO and RORγKO Th17 cells, and the whole DNA gels are shown in supplementary figure 8; **(g)** A schematic working model of TRIM28 in regulating Th17 development. **(a-e)** Those experiments were repeated 2-3 times with consistent results, and Student's t-test was used for the statistical test (ns, not significant; $p < 0.05$, *; $p < 0.01$, **; $p < 0.005$, ***). All error bars represent SDs.

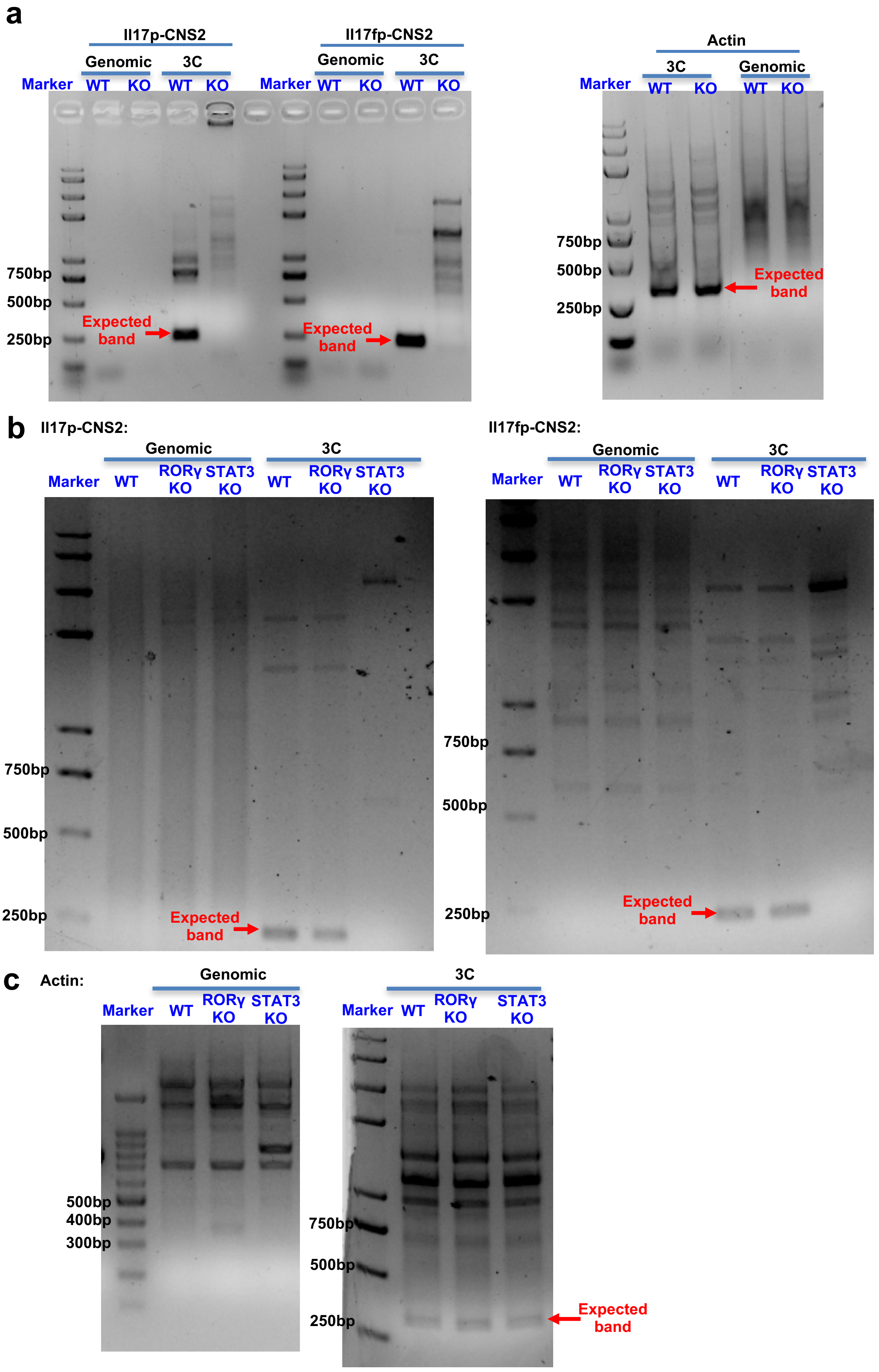

Supplemental figure 8. Whole DNA gels related to Figure 5h, 7e, supplementary figure 7e

Whole DNA gels related to **(a)** Figure 5h; **(b)** Figure 7e; **(c)** supplementary figure 7e.

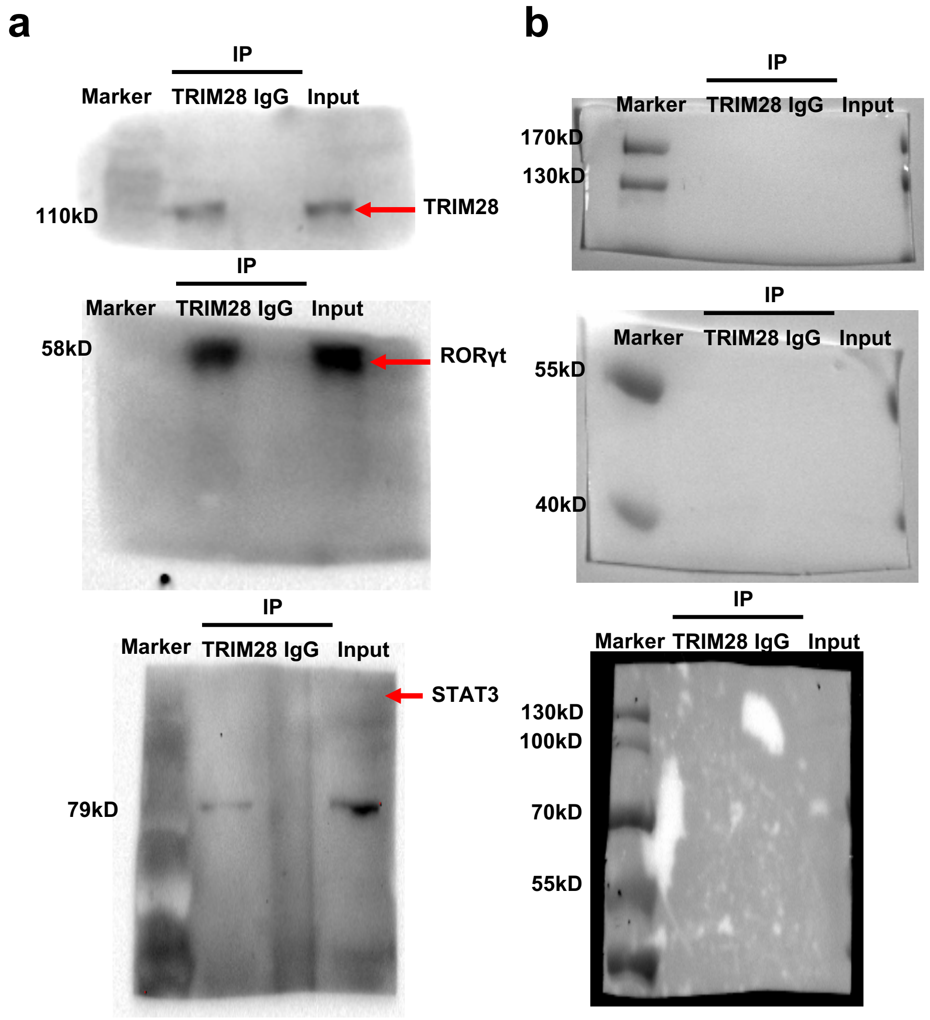

Supplemental figure 9. Whole WB gels related to Figure 7f

Whole WB gels related to Figure 7e, **(a)** up: anti-TRIM28; middle: anti-RORγt; down: STAT3; **(b)** the gel markers related to (a) were exposed in a different panel, up: anti-TRIM28; middle: anti-RORγt; down: STAT3.

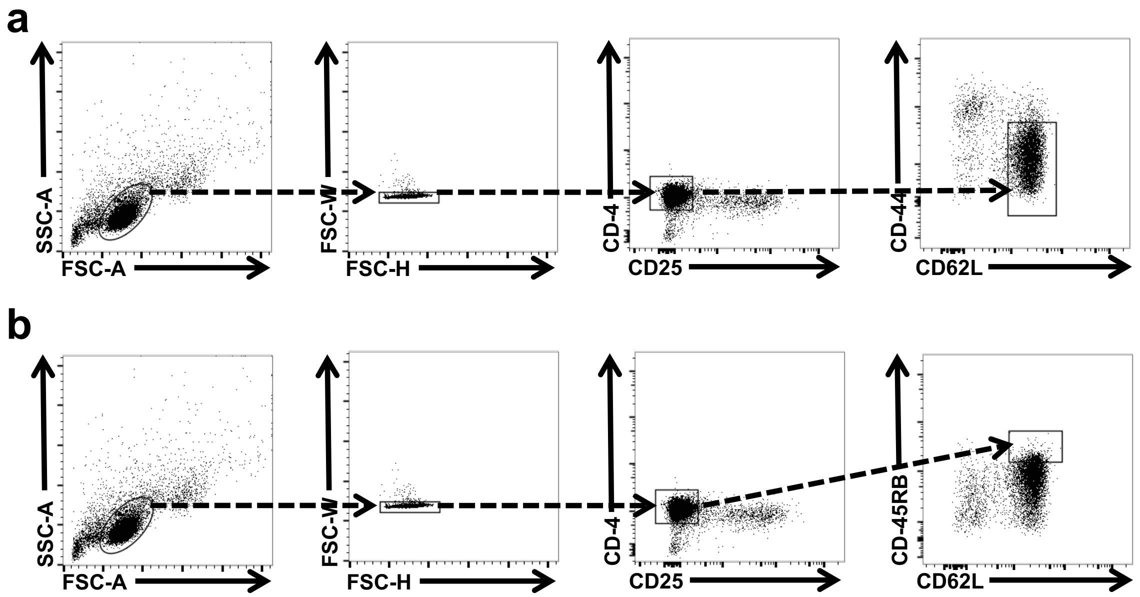

Supplemental figure 10. Gating strategies for cell sorting

(a) Gating strategy to sort CD4^{+} naive T cells (CD4^{+}CD25^{-}CD44^{low}CD62L^{hi}) from the spleen and lymph nodes; **(b)**Gating strategy to sort CD4^{+}CD25^{-}CD62L^{hi}CD45RB^{hi} T cells for colitis induction.

Supplemental table 1

Genes that are bound by TRIM28 and upregulated in *Trim28*-deficient Th17 cells

#GeneID	WT-RPKM	KO-RPKM	log_2Ratio(KO/WT)
Clec12a	0.001	5.45720064	12.41394537
Pgpep1l	3.936503859	87.35726783	4.471940938
Usp18	0.676600438	10.09354991	3.898985739
Pltp	3.580343986	27.66262861	2.949768145
Hist1h2bg	0.978045186	5.783627016	2.564001491
Acadm	30.7020574	160.0320824	2.381953916
Ramp3	1.354724751	6.753162882	2.317563596
Xcl1	2.695956228	11.59448589	2.104569872
Lgals7	13.85359231	59.5800773	2.104569872
Tbc1d30	3.211216976	12.22565779	1.928720037
Otud1	4.162202973	15.79068014	1.923654087
Sh3yl1	1.472560833	5.215440784	1.824461953
Fam188a	12.18207054	41.6497545	1.773548629
Ldhd	9.284865209	31.53999894	1.76422974
Slc1a4	1.76190132	5.930139496	1.750932918
Gstt2	34.24585956	107.8644519	1.655218008
Nars2	3.869050769	11.96507887	1.628778342
Cxcr3	6.45263487	19.80764731	1.618097235
Hilpda	2.289966204	6.89391259	1.589996699
Depdc1b	6.835202155	19.61871539	1.521174667
Shcbp1	12.46419217	35.52031261	1.510854898
Cd24a	79.92504876	223.4266254	1.483081496
Plac8	15.12386684	42.0457446	1.475132742
Hspb6	5.219069758	14.49613476	1.473803682
Hist1h2ao	140.1349422	383.7509153	1.453353459
Tesc	49.64591629	127.5236174	1.361017503
Tg	3.980604611	10.22194344	1.360610032
Tnfsf10	1.429530548	3.597219473	1.331340734
Ccnb1	37.78839741	94.10312585	1.316299311
Sema4f	23.70052146	57.44422668	1.277243103
Mypop	3.573798567	8.497736012	1.249620205
Ube2e2	12.69117677	29.92775275	1.237658106
Pdss1	3.580343986	8.389918477	1.22855859
Hmgn5	23.37059701	54.65432608	1.225641106
Ccbl2	1.80770707	4.175139149	1.207663365
Ccnf	33.23862423	76.63954755	1.205228375

Hist1h2ag	9.130896238	20.86176314	1.192032714
Chst11	3.669658424	8.300206193	1.177501395
Akr1b10	7.737387674	17.48203305	1.175954506
Parp16	4.360435044	9.441565173	1.114553961
Cenpn	8.76050231	18.92957212	1.111556303
Pxmp2	6.683308774	14.37143683	1.104569872
Hmgb2	275.220989	590.7397259	1.101932136
Kif2c	22.02667962	46.85265518	1.088878775
Fam173b	6.577269998	13.86609471	1.07600072
Slc33a1	2.311971004	4.816180583	1.058766183
Marcks	5.137015284	10.69875538	1.058440702
Gadd45b	7.14422662	14.74311192	1.045191325
Nsmce1	44.19507789	89.5060258	1.01809911
Kcnmb4	16.1739958	32.58639746	1.010593724
Tk1	10.06826754	20.13708346	1.000039289

Notes: FDR<0.01, log_2Ratio(KO/WT)>1, KO-RPKM>2

Supplemental table 2

Genes that are bound by TRIM28 and downregulated in *Trim28*-deficient Th17 cells

#GeneID	WT-RPKM	KO-RPKM	log_2Ratio(KO/WT)
Il10	2.993672001	0.001	-11.54770045
Peli2	4.370602848	0.027642104	-7.304821064
Rpph1	78.65740326	2.487364066	-4.982892969
Trim28	207.1979713	8.94075563	-4.534469301
Casr	2.016452252	0.106624751	-4.241204965
Prss12	19.78459358	1.612445444	-3.617055176
Il17f	401.2112803	47.35071238	-3.082904133
Anxa3	7.616247491	0.998632886	-2.931054037
Fhit	3.616219176	0.486008009	-2.895430128
Entpd1	1.074870961	0.154089744	-2.802320723
Gm20098	3.013882031	0.540074494	-2.480392628
Ghitm	8.860532966	1.613448474	-2.457245975
Il17a	285.852707	52.60424517	-2.442020818
Rora	23.41376429	4.390790521	-2.414804287
Furin	4.42071404	0.82989515	-2.413278433
Atrnl1	7.933642768	1.495208994	-2.407636286
Myo10	25.9042176	5.111106207	-2.341479534
Gmfg	9.943376482	1.973696818	-2.33283544
Nebl	8.376563178	1.736146731	-2.270469559

Mctp2	6.703439222	1.419210306	-2.239813073
Dgkg	3.94933796	0.849244931	-2.217358223
Ar	2.507076482	0.539108584	-2.217358223
Gja1	65.47705407	14.57971852	-2.167026547
Cyp4f39	2.469862964	0.555809006	-2.151769881
Mast4	3.410234868	0.772712199	-2.141868023
Calcrl	10.13866785	2.309210076	-2.134396774
Maf	276.5948444	63.52613087	-2.122352208
Ahr	22.3344647	5.444221495	-2.036474009
Zfyve28	5.780726245	1.421797299	-2.023534953
Cd86	7.640135375	1.910342627	-1.999766788
Myo3b	4.542962046	1.162969932	-1.965819456
Abcc3	2.134463368	0.549487664	-1.957714406
Nrip1	18.26141446	4.747905134	-1.943435501
Megf9	1.964395133	0.528016539	-1.895430128
Hivep3	52.9919486	14.58889621	-1.860902445
Fscn1	11.55859082	3.212961833	-1.846989773
Gm16675	5.586313816	1.59349707	-1.809700254
Enpp1	2.7985319	0.802375505	-1.802320723
Spin4	4.429132431	1.277633123	-1.793550514
Zfp369	6.237211308	1.803317273	-1.790247891
Sh3bp5	30.77627412	9.231351158	-1.737204854
Trp53inp1	1.38466342	0.416851068	-1.731931395
Iglon5	14.93785901	4.534812555	-1.719858563
Syne1	4.894497461	1.516294939	-1.690610336
Maml2	10.95427786	3.481473987	-1.653724231
Gipr	3.608315423	1.172892096	-1.621255164
Tns4	2.087216305	0.680036443	-1.617896152
Prrg4	7.978405326	2.613218046	-1.610272911
Pdpn	21.76136718	7.128850094	-1.610027909
Hipk2	4.978351041	1.632350919	-1.608716725
Alpk2	23.41693119	7.69367437	-1.605807347
Foxn3	25.57114311	8.421408788	-1.602383153
Ptpn4	3.386380163	1.133502427	-1.578956463
Cbl	23.16411417	7.792892535	-1.571660686
Adora2a	23.32168817	7.847859396	-1.571301125
Mansc1	3.563968192	1.210069005	-1.55839514
Cdk6	26.90911164	9.425800672	-1.513407686
Tram2	5.300969729	1.861049373	-1.510139972
Fam20a	15.99238576	5.69029729	-1.490809244
Lnpep	25.74447691	9.174585467	-1.488548077
Lyst	4.725471793	1.725521913	-1.453425581
Satb1	6.501909198	2.398263286	-1.438873359
Dusp16	2.9121812	1.075327579	-1.437323907

Tbkbp1	5.463276529	2.022186986	-1.433850043
Il3ra	21.57452617	8.002745937	-1.430761861
Ston2	4.786575478	1.780817948	-1.426453821
Zfp58	4.76874569	1.777439861	-1.4238091
Il21	173.1362046	64.89251077	-1.415783547
Alcam	8.788705402	3.312155806	-1.407880129
Sntb1	11.73381661	4.43058114	-1.405104501
Ptger2	18.59726446	7.208602867	-1.36729885
Paqr8	9.114629643	3.570191325	-1.352182646
Kctd12	8.662122547	3.406002145	-1.34664124
Mdn1	12.74402299	5.032483621	-1.340478298
Rgs1	112.6408742	44.78366576	-1.330685906
Heg1	8.263708184	3.298190904	-1.325114403
Nedd4	35.8282038	14.30211701	-1.324867006
Tex15	4.381748289	1.749852337	-1.324273427
Scaper	3.974529034	1.598648135	-1.313931482
Nxpe3	63.1198497	25.42101549	-1.312072107
Malt1	146.2607694	59.07986335	-1.307804462
Fnip1	14.91534481	6.100595994	-1.289775232
Myo1h	2.045689956	0.837894179	-1.287747551
Smox	6.727277911	2.7655561	-1.282453251
Obsl1	2.442577857	1.005777072	-1.280093978
Elk4	15.36105088	6.342066919	-1.276251912
Slc26a2	6.072589644	2.511188159	-1.273941751
Arhgef9	4.440426619	1.838723836	-1.271993479
Dmxl1	4.950497736	2.052263776	-1.270357414
Rasa1	45.51620208	18.95389948	-1.263885491
Ube2q2	84.20357777	35.21659253	-1.25762621
Nsg2	33.85603921	14.20004319	-1.253517886
Ly75	8.344444394	3.563682856	-1.22744704
Tnfsf8	17.27821299	7.401805996	-1.223004786
Ptch1	10.93314798	4.694502448	-1.21966469
Klf7	15.32834769	6.616314387	-1.212102497
Adam19	50.42299098	21.79169223	-1.210303465
Smo	23.44283565	10.16335581	-1.205770248
Mgat5	6.007226636	2.604359928	-1.205770248
Mib1	8.246212777	3.580545162	-1.203552423
H2-T24	4.755195633	2.0747033	-1.196599662
Zfp677	4.950599092	2.162368967	-1.19499041
Rapgef2	3.975363601	1.754154212	-1.180311236
BC016423	10.11374335	4.508272951	-1.165670303
C1galt1	10.75134005	4.810017844	-1.160402338
Eif5	5.080730195	2.282906001	-1.154164396
Arap1	3.890104773	1.772731628	-1.133834867

Dusp6	30.88714663	14.07650062	-1.133717871
Ptgfrn	43.55817039	19.8675303	-1.132530812
Acvr2a	5.902746307	2.732116137	-1.111367527
Lysmd3	5.892340564	2.729840461	-1.110024179
Zfyve16	3.641428556	1.690640959	-1.106934233
Lamc1	29.00163182	13.53332299	-1.099617954
Farp1	11.7958955	5.525768638	-1.094037884
Tmtc2	2.29478634	1.077668697	-1.09044611
Prkacb	2.840521697	1.340804403	-1.083057131
Pdgfb	28.4877167	13.45499033	-1.082198639
Rel	11.231528	5.318377115	-1.078496229
Slco3a1	9.572484517	4.543662697	-1.075037683
Baz2b	6.161960439	2.937770216	-1.068667863
Raph1	19.34728823	9.239287496	-1.066277863
Arap2	14.52074319	6.938809985	-1.065355129
Myb	3.662814181	1.755050367	-1.061440079
Egr3	37.16882528	17.81205624	-1.061239021
Zfp507	10.59038185	5.09015192	-1.056973988
Rasgrp1	22.92809227	11.0713033	-1.050291257
Smad3	38.96736663	18.86780483	-1.046339856
Ammecr1	7.06164983	3.432226389	-1.040860567
Dopey1	4.647697739	2.26817942	-1.03498148
Med13l	21.05080831	10.2752302	-1.034704916
Hspa4l	25.0624079	12.25249386	-1.032449607
Cxcr4	41.45413103	20.3319133	-1.027769894
Tab3	3.770555021	1.849638379	-1.027533664
Stat1	6.353849891	3.119403131	-1.026360998
Bmp2k	9.887947719	4.861408585	-1.024296722
Pde3b	53.39792666	26.28723484	-1.022421333
Bend4	2.937371058	1.446652195	-1.021807413
Stat4	26.2814784	12.96249606	-1.019702882
Chm	36.08739564	17.97167812	-1.005769903
Ubtd2	6.052374683	3.015603359	-1.005054619
Mxi1	5.278854729	2.634020132	-1.002958592
Zbtb26	2.814190982	1.405901429	-1.001224792

Notes: FDR<0.01, log_2Ratio(KO/WT)<-1, WT-RPKM>2

Supplemental table 3

Primers used in ChIP- and MeDIP-qPCR

Name	Forward	Reverse
IL17p	CAGCTCCCAAGAAG TCATGC	TGAGGTCAGCACAGAACCAC
IL17CNS2	TGTGGTTGTCTAAGCCATGC	CAGCAACTGACTGGGTTTCA
Il7CNS2-0	ATGGGCCTCTCTTTCCACTGATG	GGAATTTGTGGTGGAAGGGAGTG
IL17CNS3	GCACTTTGCTCATGCCCATAT	TGAACCAACTTTCCCCACTCT
IL17fp	TCCCTGTTTCCACTGACCTC	TTAGGGTCCCCCTTTGATTC
Ifngp	GGGGGAGACGTAAAAGCAAT	GGGCTCTCTGACGATGAGAC
H19	GCATGGTCCTCAAATTCTGCA	GCATCTGAACGCCCCAATTA

Supplemental table 4

Primers used in 3C assay

Name	Sequence
CNS2-5'end-1	TTTTCCCTAGCAGTGGTTTTGGA
CNS2-5'end-2	GAGGTTGATGGCAGAGCTCA
IL17p-3'end-1	TGATACCGAACCTCAAAACAGCA
IL17p-3'end-2	CTCTCTAGCCAGGGAATTTGGT
IL17fp-3'end-1	CCTGCAAGACTGGAAAGGAGAAA
IL17fp-3'end-2	AAGACAAGCTCAGAAAAGGCC
Actin-1	AGCAGTGGTTTCTATTGGCTGTCG
Actin-2	CAAGCAGGATTCCTGTTCCTT
