# Supplementary material for: Epigenetic activation during T helper 17 cell differentiation is mediated by Tripartite motif containing 28
Source: Nat Commun. 2018 Apr 12;9:1424. doi: 10.1038/s41467-018-03852-2 (PMC5897371; doi:10.1038/s41467-018-03852-2)
Supplement: Supplementary file 2 — Peer Review File [file 41467_2018_3852_MOESM2_ESM.pdf]

Reviewers' comments:

Reviewer #1 (Th17, cytokine signaling)(Remarks to the Author):

Comments to the author

This study by Yu Jiang et al. describes a molecular mechanism proposed for the epigenetic activation for Th17 cell differentiation, and focus on the role of STAT3-TRIM28 axis in Th17 cells. Jiang et al. demonstrated the critical role of TRIM28 during Th17 cell differentiation in vivo and in vitro by the epigenetic analysis. This study is interesting and supported by experimental data. However, several aspects remain unclear.

Major remarks:

1. The authors showed that TRIM28 acts as a transcription factor during Th17 cell differentiation. Regarding to this, they should compared the localization of TRIM28 during Th17 cell differentiation.

2. The authors described that STAT3 controls the transcription of Trim28. Several cytokines and its receptor induce STAT3 transcription activity. It helps to better understanding which cytokines induce TRIM28 expression during Th17 cell differentiation.

3. In Figure 3b, Jiang et al showed EAE mouse model which pathogenesis is dependent on IL-17 production derived Th17 cells. It should be better to add the experiment by adoptive transfer of CD4 positive T cells during EAE, and compare Trim28 positive and negative CD4 T cells.

Reviewer #2 (IRF4, Th1/Th17)(Remarks to the Author):

The manuscript by Jiang et al focuses on the mechanisms how TRIM28 regulates Th17 differentiation. Previously published data by Chikuma et al, NI 13: 596, 2012, reveal a negative impact of TRIM28 on Th17 differentiation. In the cited paper, autoreactive Th17 cells accumulated in TRIM28 CKO mice and consequently TRIM28 deficiency exacerbated EAE. Furthermore, TRIM28 was shown to impact Treg differentiation, and the disturbed function of Treg was visible in the loss of protective function in the transfer model of colitis. The CKO mice were defective in IL-2 production and IL-2 response in vivo. In opposite to these published data, Jiang et al demonstrate, that TRIM28 positively regulates Th17 differentiation. The positive function of TRIM28 on the Th17 differentiation and function is rather convincing, however it will be interesting to get more information on the expression and function of TRIM28 in human Th17 cells in light of treatment of autoimmunity. Concerning molecular mechanisms I have the following questions:

1. SE are characterized by occupation with several transcription factors enabling transcription. IRF4 and its partner BATF promote the initial Th17 differentiation together with STAT3, are they needed for the function of TRIM28?
2. SE are known to associate with H3K27Ac, how does TRIM28-deficiency affect this modification at SE genome-wide?
3. Does TRIM28 deficiency cause less RNA pol II recruitment preferentially to Th17-signature genes?
4. The authors analyze the effects using TRIM28-deficient cells, can the effects be rescued by overexpression or gradually downregulated by shRNA usage in WT cells?
5. How does TRIM28 negatively impact on Foxp3 expression?

Further questions:

1. In 1a the authors show the percentages of the cells, what about the absolute numbers of CD4+ naïve versus memory cells in LNs of WT and TRIM28CKO mice? Please, show the proliferation profiles of the cells in vitro. 1d, what about the numbers of transferred WT and KO cells at the day of analysis? Do the KO cells acquire Foxp3 expression in vivo? Please use another statistical test, not t-test, for 1b. For which time-points are the differences significant using the appropriate statistical test (Anova?)?
2. In Fig 2, Foxp3 is upregulated in TRIM28 deficient cells, what about other genes associated with Treg signature? 2e,it is difficult for me to understand the heatmap of pathway analysis, are WT on the left and KO cells on the right side? Please show statistic for 2a.
3. Please show statistic for 3a, IL-17 and Foxp3. In 3d please show numbers of infiltrating CD4 and CD8 T cells. 3b please, do not use t-test for the EAE statistical analysis, how many animals were used in the experiment? For which time-point is the significance shown? What happens after the first immunization (show the data)?
4. Does TRIM28 impact Csf2 regulating regions?
5. 5d there is no description at the x-axis, what is the median size? How many bp? 5e, please describe better the heatmap of pathways analysis, actually Th17 signature genes are not listed. 5f,g, what is the statistical test used?
6. Fig 6e, in my opinion RORgt rescued the phenotype, but partially. May be there is a need for RORa? Please show statistics for 6e.
Please show statistic for 7c.7d, there is no description at the x-axis, what is the median size? How many bp? 7f, why is the input on a different gel? Please repeat the immunoblotting with all controls on one gel.

Point-by-point responses to the reviewers' comments

**We thank the reviewers for their comments and the opportunity to revise our manuscript. We outline our responses in details below.**

## Reviewer #1

*This study by Yu Jiang et al. describes a molecular mechanism proposed for the epigenetic activation for Th17 cell differentiation, and focus on the role of STAT3-TRIM28 axis in Th17 cells. Jiang et al. demonstrated the critical role of TRIM28 during Th17 cell differentiation in vivo and in vitro by the epigenetic analysis. This study is interesting and supported by experimental data. However, several aspects remain unclear.*

*Major remarks:*

*1. The authors showed that TRIM28 acts as a transcription factor during Th17 cell differentiation. Regarding to this, they should compare the localization of TRIM28 during Th17 cell differentiation.*

**Response: We wish to point out that TRIM28 is not a transcription factor, but rather a co-factor which does not bind to DNA directly. To address the reviewer's comment, we performed immunofluorescence analysis on TRIM28 protein in naive T cells and Th17 cells at different time points (4h, 12h, 24h, 48h, 72h) of differentiation. We found that TRIM28 mainly localizes in the nuclei in both naive and differentiating Th17 cells, as shown below. We have discussed about this point in the revised manuscript (Page 15).**

**Figure 1**

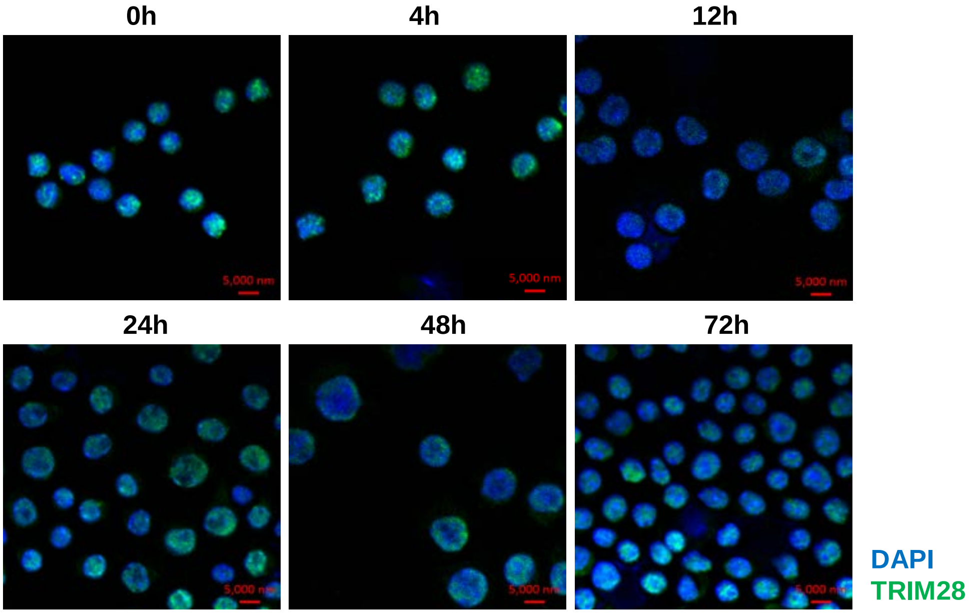


Figure 1. Naive $CD4^+$ T cells were isolated and cultured in the presence TGF-β and IL-6 for indicated time, and then fixed for immunofluorescence staining. The merged graphs were shown here, with TRIM28 protein in green, nuclei in blue and scale bars (5000nm) in red.

*2. The authors described that STAT3 controls the transcription of Trim28. Several cytokines and its receptor induce STAT3 transcription activity. It helps to better understanding which cytokines induce TRIM28 expression during Th17 cell differentiation.*

**Response: We thank the reviewer for this important question. We thus compared TRIM28 expression level in different CD4$^+$ T cell subsets and found that its mRNA was already high in naïve CD4$^+$ T cells, which was not significantly changed in any T cell subset, as showed in the new Figure 7a of the revised manuscript. It's likely that as a scaffold protein, TRIM28 is constantly highly expressed and its function rather than expression is tightly regulated in different T cell types, which supports our idea on the STAT3-regulated recruitment of TRIM28 being a critical step in T cell differentiation.**

*3. In Figure 3b, Jiang et al showed EAE mouse model which pathogenesis is dependent on IL-17 production derived Th17 cells. It should be better to add the experiment by adoptive transfer of CD4 positive T cells during EAE, and compare Trim28 positive and negative CD4 T cells.*

**Response: We appreciate the comment from the reviewer but argue respectfully that transfer of CD4 positive T cells in EAE may not be very conclusive when interpreting the results, as TRIM28 does have important function in Treg and Th1 cells. This is why we induced EAE in the *Trim28$^{f/f}$Il17fcre* mice, in which TRIM28 was specifically deleted in Th17 cells, and we found ameliorated EAE phenotype in the KO mice (Figure 3b). In addition, intracellular staining results showed no gross difference in IFNγ-producing Th1 cells or Treg cells, but only defects in Th17 differentiation and its related cytokine production in vivo (Figure 3c, 3d). Thus, our experimental evidence has sufficiently proved that TRIM28 can promote Th17-mediated autoimmune pathogenesis in vivo.**

## Reviewer #2

*The manuscript by Jiang et al focuses on the mechanisms how TRIM28 regulates Th17 differentiation. Previously published data by Chikuma et al, NI 13: 596, 2012, reveal a negative impact of TRIM28 on Th17 differentiation. In the cited paper, autoreactive Th17 cells accumulated in TRIM28 CKO mice and consequently TRIM28 deficiency exacerbated EAE. Furthermore, TRIM28 was shown to impact Treg differentiation, and the disturbed function of Treg was visible in the loss of protective function in the transfer model of colitis. The CKO mice were defective in IL-2 production and IL-2 response in vivo.*
*In opposite to these published data, Jiang et al demonstrate, that TRIM28 positively regulates Th17 differentiation. The positive function of TRIM28 on the Th17 differentiation and function is rather convincing, however it will be interesting to get more information on the expression and function of TRIM28 in human Th17 cells in light of treatment of autoimmunity.*

*Concerning molecular mechanisms I have the following questions:*
*1. SE are characterized by occupation with several transcription factors enabling transcription. IRF4 and its partner BATF promote the initial Th17 differentiation together with STAT3, are they needed for the function of TRIM28?*

**Response: We followed the reviewer's suggestion and analyzed TRIM28 recruitment in differentiating Th17 cells in which the Irf4 or Batf gene was knocked down. We found a decrease of TRIM28 recruitment in those cells, which suggests that these two factors may work with STAT3 to regulate TRIM28 function. We have added the data to Supplemental Figure 7b-c in the revised manuscript.**

*2. SE are known to associate with H3K27Ac, how does TRIM28-deficiency affect this modification at SE genome-wide?*

**Response: As the reviewer suggested, we performed H3K27Ac ChIP-seq in WT and *Trim28$^{fl/fl}$Cd4cre* Th17 cells. As shown in the new Supplemental Figure 5c-e, TRIM28**

**deficiency resulted in a preferential decrease in H3K27Ac in Th17-specific super-enhancers, which are associated with many Th17 signature genes and positive regulators. This result further supports a critical role of TRIM28 in regulation of super-enhancer establishment in Th17 cells.**

*3. Does TRIM28 deficiency cause less RNA pol II recruitment preferentially to Th17-signature genes?*

**Response: Thanks for the comment. We thus analyzed PolII recruitment at the *Il17-Il17f* locus in WT and *Trim28*$^{fl/fl}$*Cd4cre* Th17 cells and found a significant decrease in the KO cells, as shown below, thus supporting our conclusion that TRIM28 plays an important role in regulating transcription of Th17 signature genes. This result has been added in the revised manuscript (Supplemental Figure 4c).**

**Figure 2**

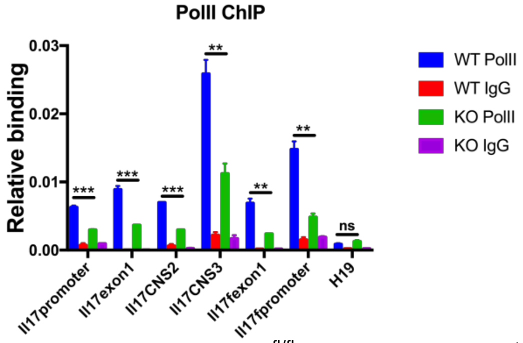


Figure 2. WT or Trim28$^{fl/fl}$Cd4cre naive CD4$^+$ T cells were cultured at Th17 condition (TGF-β plus IL-6) for 3 days, and then prepared for ChIP experiment using anti-phospho-PolII(Ser5) antibody (CST, #13523). This experiment was repeated twice with consistent results.

*4. The authors analyze the effects using TRIM28-deficient cells, can the effects be rescued by overexpression or gradually downregulated by shRNA usage in WT cells?*

**Response: We conducted the overexpression experiment as the reviewer requested. As shown below, overexpression of TRIM28 could rescue the Th17 differentiation defect by up-regulating IL-17 expression and down-regulating FOXP3 level in *Trim28*$^{fl/fl}$*Cd4cre* cells. Although TRIM28 overexpression hardly had any effect in WT cells, it is possible that as a co-activator and epigenetic regulator, the endogenous high expression of TRIM28 (Figure 7a) is sufficient for epigenetic activation and chromatin accessibility in Th17 cells.**

**Figure 3**

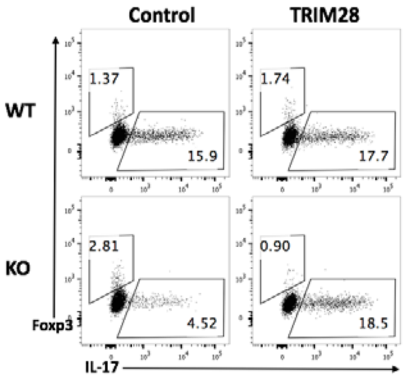


Figure 3. Overexpression of TRIM28 in WT or Trim28$^{fl/fl}$Cd4cre CD4$^+$ T cells polarized under Th17 condition (TGF-β plus IL-6) for 3 days. This experiment was repeated for 3 times with consistent results.

*5. How does TRIM28 negatively impact on Foxp3 expression?*

**Response: In our ChIP-seq study, we identified a single TRIM28 binding peak at the Foxp3 gene locus in Th17 cells, which is not located in the promoter or *Foxp3* gene regulatory regions CNS1-3 (Reference: Ye, Z. *et al.* Role of conserved non-coding DNA elements in the Foxp3 gene in regulatory T-cell fate. *Nature* 463, 808-812 (2010)). Moreover, this single peak is not co-localized with active epigenetic modifications H3K4Me3 or 5hmc, suggesting that TRIM28 might regulate Foxp3 gene expression through an indirect manner. We have added this discussion on Page 21 of the revised manuscript.**

**Figure 4**

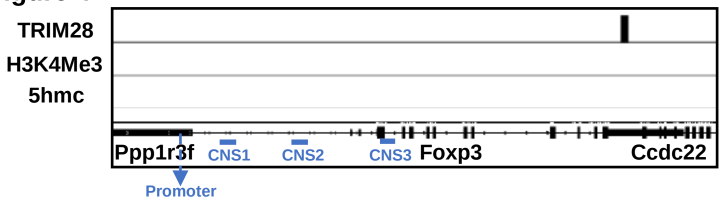


Figure 4. IGV browser view of histone marker-H3K4me3 and DNA marker-5hmc together with TRIM28 binding peaks at the Foxp3 gene locus in WT Th17 cells cultured in vitro.

*Further questions:*
*1. In 1a the authors show the percentages of the cells, what about the absolute numbers of CD4+ naïve versus memory cells in LNs of WT and TRIM28CKO mice?*

**Response: We compared the absolute numbers of CD4$^+$ naive versus memory cells in the peripheral lymph nodes of WT and TRIM28KO mice, but did not find any significant difference. We have added this discussion on Page 6.**

**Figure 5**

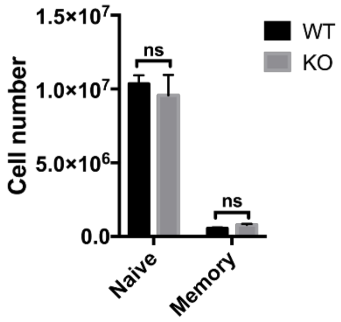


Figure 5. Cell number of naive (CD4$^+$CD25$^-$CD62L$^{hi}$CD44$^{lo}$) and memory (CD4$^+$CD25$^-$CD62L$^{lo}$CD44$^{hi}$) CD4$^+$ T cells in the peripheral lymph nodes of WT (n=4) and Trim28$^{fl/fl}$Cd4cre mice (n=3) (7~8 weeks old mice).

*Please, show the proliferation profiles of the cells in vitro.*

**Response: We addressed the reviewer's comment by testing the proliferation ability of *Trim28$^{fl/fl}$Cd4cre* cells under different TCR stimulation *in vitro*. We found that KO cells only showed obvious defect of proliferation under low dose of anti-CD3/CD28 stimulation. Importantly, we observed obvious Th17 differentiation defects in the KO cells under any TCR stimulation tested. In addition, in the colitis experiment, there is no proliferation defect in the KO cells *in vivo* (seen in the result for the next question), thus there might not be a proliferation defect in KO cells to contribute to its Th17 differentiation defect either *in vitro* or *in vivo*. We have added this discussion on Page 20.**

**Figure 6**

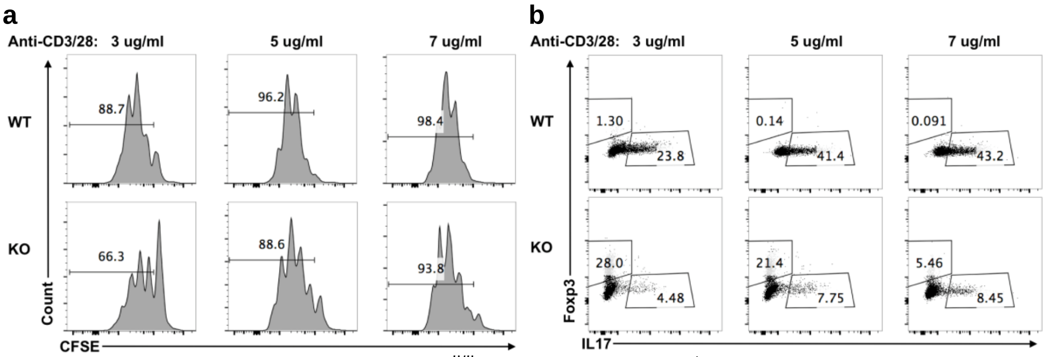


Figure 6. CFSE-labeled WT or Trim28$^{fl/fl}$Cd4cre naive CD4$^{+}$ T cells were cultured with indicated concentrations of anti-CD3&CD28 stimulation in the presence of TGF-β and IL-6 for 3 days, and then harvested for CFSE detection(a) and intracelluar staining(b). This experiment was repeated for twice with consistent results.

*1d, what about the numbers of transferred WT and KO cells at the day of analysis? Do the KO cells acquire Foxp3 expression in vivo?*

**Response: We compared the numbers and proliferation ability of transferred WT and TRIM28KO cells at the day of analysis but did not observe any obvious difference. Very few WT and KO cells acquired Foxp3 expression *in vivo* (less than 3% in CD4$^{+}$ T cells). In addition, there was no significant difference in the percentages or cell numbers. Treg cells may not contribute to the phenotype. Part of these data have been added in the Supplemental Figure 1c in the revised manuscript.**

**Figure 7**

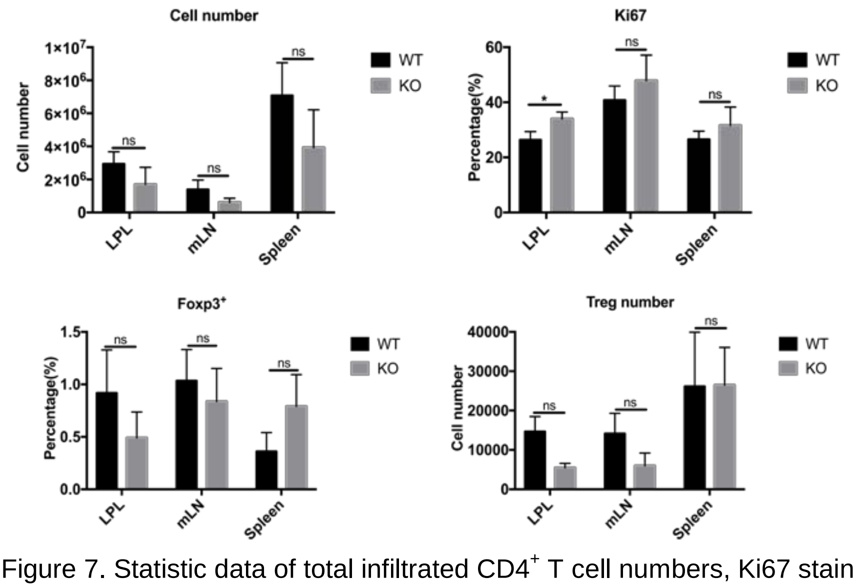


Figure 7. Statistic data of total infiltrated CD4$^{+}$ T cell numbers, Ki67 staining in CD4$^{+}$ T cells, and Treg percentage and cell numbers in the lamina propria (LPL) of large intestine, mesenteric lymph nodes (mLN) and spleens isolated from colitis mice.

*Please use another statistical test, not t-test, for 1b. For which time-points are the differences significant using the appropriate statistical test (Anova?)?*

**Response: The statistical test used for 1b is two-way-ANOVA. According to this test, the 2 groups started to show significant difference 2 weeks after transfer. We have added a statement for the statistical test in the figure legends.**

*2. In Fig 2, Foxp3 is upregulated in TRIM28 deficient cells, what about other genes associated with Treg signature? 2e,it is difficult for me to understand the heatmap of pathway analysis, are WT on the left and KO cells on the right side? Please show statistic for 2a.*

**Response: In response to the reviewer's comment, we have changed the labeling for Figure 2e and included statistic test for Figure 2a in the revised manuscript.**
**In addition, we analyzed some Treg signature genes and found most of them showed mild or no increase in TRIM28-deficient Th17 cells. This is discussed in the revised manuscript on Page 7.**

**Figure 8**

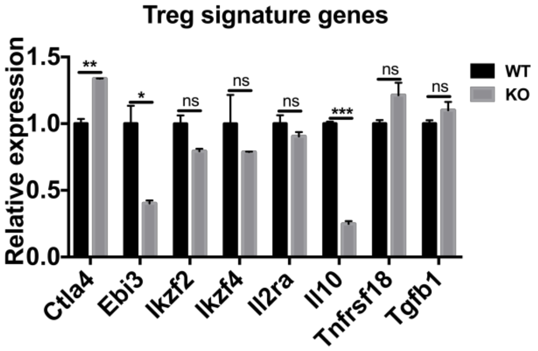


Figure 8. WT or TRIM28-deficient naive CD4$^+$ T cells were polarized into Th17 cells in the presence of TGF-β and IL-6 for 3 days, and then re-stimulated for mRNA analysis by real-time RT-PCR assay. This experiment was repeated for twice with consistent results.

*3. Please show statistic for 3a, IL-17 and Foxp3. In 3d please show numbers of infiltrating CD4 and CD8 T cells. 3b please, do not use t-test for the EAE statistical analysis, how many animals were used in the experiment? For which time-point is the significance shown? What happens after the first immunization (show the data)?*

**Response: To address the reviewer's comment, we have included a statistic test for 3a and showed infiltrated CD4$^+$ T cell numbers in the revised manuscript. Two-way-ANOVA was used figure 3b and added in the figure legends. The animal numbers used in EAE were shown in the figure legends. The significance was shown at 12 days after 2$^{nd}$ immunization. According to the protocol (seen reference paper in the method), the mice started to show disease symptom only after the second immunization but not the first immunization.**

*4. Does TRIM28 impact Csf2 regulating regions?*
**Response: As shown below, no TRIM28 binding peak was seen at the *Csf2* locus. We have added this discussion on page 10.**

**Figure 9**

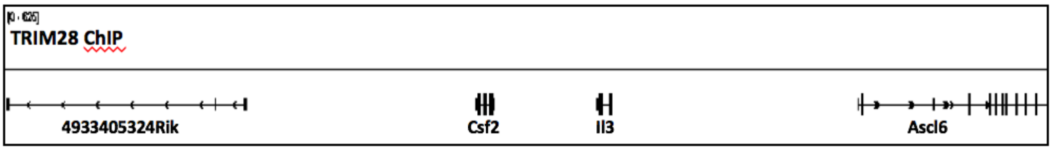


Figure 9. IGV browser view of TRIM28 binding peaks at the *Csf2* locus in WT Th17 cells cultured *in vitro*.

*5. 5d there is no description at the x-axis, what is the median size? How many bp? 5e, please describe better the heatmap of pathways analysis, actually Th17 signature genes are not listed. 5f,g, what is the statistical test used?*

**Response: We have labeled the x-axis of 5d and listed the Th17 signature genes in supplemental table 5 in the revised manuscript. T-test was used in the figure 5f, 5g.**

*6. Fig 6e, in my opinion RORgt rescued the phenotype, but partially. May be there is a need for RORa?*

**Response: We changed our description more precisely in the revised manuscript. In addition, when we overexpressed RORα together with RORγt, RORα could not further enhance the effect of RORγt in TRIM28 KO T cells, despite itself can also partially rescued the phenotype, under both in Th0 and Th17 condition (data shown below), indicating TRIM28 may regulate Th17 differentiation through additional mechanism other than ROR factors. We have discussed about it in the revised manuscript (Page 14-15).**

**Figure 10**

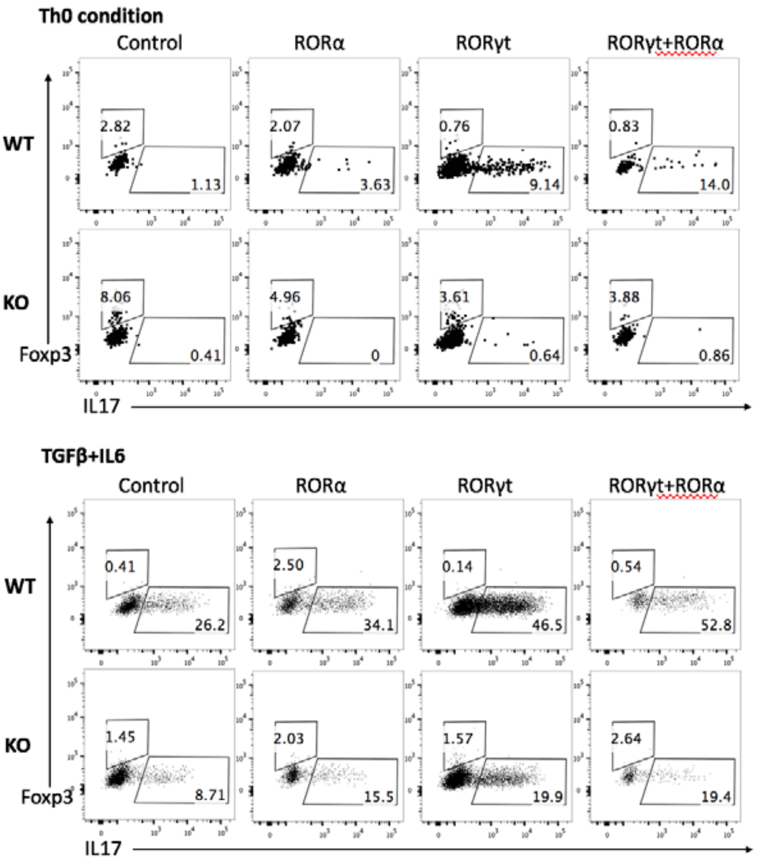


Figure 10. Overexpression of RORγt and RORα alone or together in WT and TRIM28 KO $CD4^+$ T cells polarized under neutral (Th0) or Th17 (TGF-β plus IL-6) conditions. This experiment was repeated twice with consistent results.

*Please show statistics for 6e. Please show statistic for 7c, there is no description at the x-axis, what is the median size? How many bp?*

**Response: The median of x-axis is the normalized SE region, with 20kb up- and down-stream of SE regions. We have included the information in the revised manuscript.**

*7f, why is the input on a different gel? Please repeat the immunoblotting with all controls on one gel.*

**Response: We have repeated this experiment and the result is shown in the revised manuscript (Figure 7f).**

Reviewers' comments:

Reviewer #1 (Remarks to the Author):

This revised manuscript has almost completed to both reviewer`s suggestions and comments. I agree this manuscript is appropriate for nature communications.

Reviewer #2 (Remarks to the Author):

In this paper, Jiang et al use aCD4 cre directed konditional TRIM 28 ko mouse to contradict previous findings generated with an lck-directed konditional TRIM 28 ko in that they show a positive efffect of TRIM 28 on Th17 differentiation. They also show that, unexpectedly, TRIM 28 under these conditions is more a stimulatory than a suppressive factor. I find the manuscript interesting and also improved. I would like to see two more controls and otherwise corrections from annotation mistakes which seem to indicate a rather rushed preparation of the manuscript. Likewise, the English language should be corrected by a native speaking person.
Controls:
1)Since in contrast to the competing paper, the mice under analysis show no phenotype with respect to CD4/CD8 cell appearance in the periphery, I would like to see a PCR to test for appropriate excision of TRIM 28.
2) In Fig. 4, only very few signals are seen positive for H3K27Me3. Please show data for at least two appropriate and suppressed control genes.

Corrections in the text
In sFig. 2c, STAT vs Stat
In Fig 2b and sFig. 2c, I do not see IRF4 as is stated in the text on p7
P 8, 2nd chapter. TRIM28 deficiency does not lead to increased Foxp3 expression in Th17 cells. It rather seems to lead to more Treg cells under Th17 conditions, because the IL17+ cells do not have enhanced Foxp3 (Fig. 2a)
sFig. 3, annotations of b and c are switched, how were pStat molecules measured? FACS? The Figure only shows percentages, not levels as stated.
P 9, 1st chapter. Anti-Il-2 DOES rescue IL17 production by 6-fold (sFig 3d)
P 16, end of 1st paragraph, s Fig. 7b,c instead of s Fig. 6
P 16 2nd paragraph, there is no s Fig. 6 d.

Responses to reviewers' critiques

**We thank the reviewers for their comments. We outline our responses in details below.**

Reviewer #1

*This revised manuscript has almost completed to both reviewer's suggestions and comments. I agree this manuscript is appropriate for nature communications.*

**Thanks a lot.**

Reviewer #2:

*In this paper, Jiang et al use a CD4 cre directed conditional TRIM28 ko mouse to contradict previous findings generated with an lck-directed conditional TRIM28 ko in that they show a positive effect of TRIM28 on Th17 differentiation. They also show that, unexpectedly, TRIM28 under these conditions is more a stimulatory than a suppressive factor. I find the manuscript interesting and also improved. I would like to see two more controls and otherwise corrections from annotation mistakes which seem to indicate a rather rushed preparation of the manuscript. Likewise, the English language should be corrected by a native speaking person.*
*Controls:*
*1) Since in contrast to the competing paper, the mice under analysis show no phenotype with respect to CD4/CD8 cell appearance in the periphery, I would like to see a PCR to test for appropriate excision of TRIM28.*

**Response: We thank the reviewer for this important question. We thus analyzed the deletion efficiency of the *Trim28* gene in peripheral $CD4^+$ and $CD8^+$ T cells and thymic $CD4^+$ single positive(SP)/$CD8^+$SP/$CD4^+CD8^+$(DP) T cells in *Trim28*$^{f/f}$*CD4cre* mice. Real-time PCR analysis found that in KO mice, more than 50% of *Trim28* genomic DNA was excised at the DP stage, very little remained in the thymic $CD4^+$/$CD8^+$ SP T cells, and undetectable in the peripheral T cells (Figure 1A). In accordance with this, the TRIM28 protein levels were barely affected in the DP stage, slightly lower in the $CD4^+$ SP stage and undetectable in the peripheral CD4+ T cells (Figure 1B). Since *Trim28* deletion by *Lck-cre* occurs in the $CD4^-CD8^-$ (DN) stage, much earlier than using *Cd4-cre*, and TRIM28 is highly expressed in the thymus (Figure 1B), we believe the alteration of CD4/CD8 numbers in the *Trim28* $^{f/f}$*Lck-cre* mouse might implicate a potential function of TRIM28 during thymus development.**
**In addition, different housing environments and microbiota may also contribute to our difference. By 20-week of age, the percentages of naive T cells in our WT mice are higher than those in the Honjo's group (76.8% vs 64%). Although not significant, the $CD4^+$ T cells in our *Trim28*$^{f/f}$*CD4cre* mice became more activated at an older age (Figure 1a, Supplementary Fig. 1a in the manuscript). Since TRIM28 deletion restrains effector T cell differentiation and impairs Treg function, it's likely the peripheral T cell became activated in the *Trim28* $^{f/f}$*Lck-cre* mice in a cell-extrinsic way.**
**We have added this discussion into the revised manuscript (page 6).**

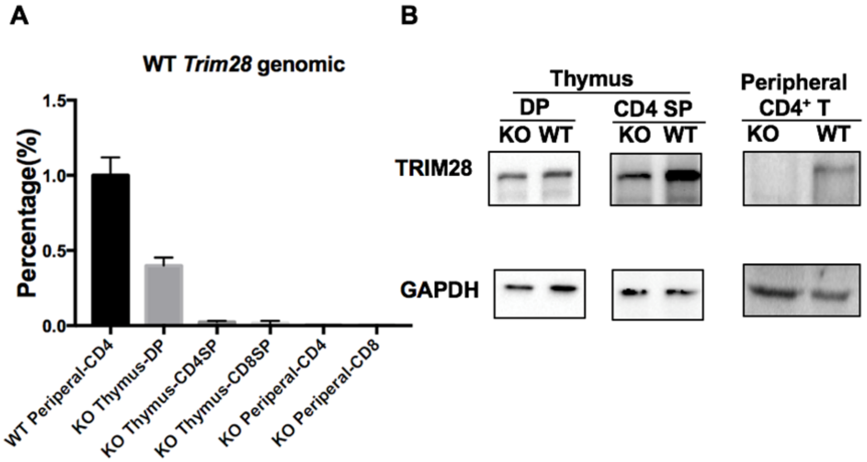


**Figure 1 (A) CD4$^+$CD8$^-$ T cells, CD8$^+$CD4$^-$ T cells and CD4$^+$CD8$^+$ T cells in the thymus, as well as CD4$^+$ T cells and CD8$^+$ T cells in the peripheral (spleen and lymph nodes) were sorted from KO (*Trim28$^{f/f}$CD4cre)* mice, and genomic DNA was extracted for real-time PCR to analyze excision of *Trim28* genomic DNA. Peripheral CD4+ T cells from wild-type (WT) animals were used as control. The result shows the percentages of WT *Trim28* left in each cell population from KO mice (normalized to genomic *actin*); (B) WB analysis of TRIM28 and GAPDH in DP and CD4SP T cells in the thymus, as well as CD4$^+$ T cells in the peripheral sorted from WT or KO (*Trim28$^{f/f}$CD4cre)* mouse.**

*2) In Fig. 4, only very few signals are seen positive for H3K27Me3. Please show data for at least two appropriate and suppressed control genes.*

**Response: Following the reviewer's suggestion, we examined the H3K27Me3 ChIP-seq data and found many peaks at the suppressed genes (*Tbx21, Gata3, Ifng, Gzmb*) in Th17 cells, as shown below. This indicates both the quality and processing of the H3K27Me3 ChIP-seq data are good. We have added the *Tbx21* locus as a positive control for H3K27Me3 in Supplementary Fig. 4b.**

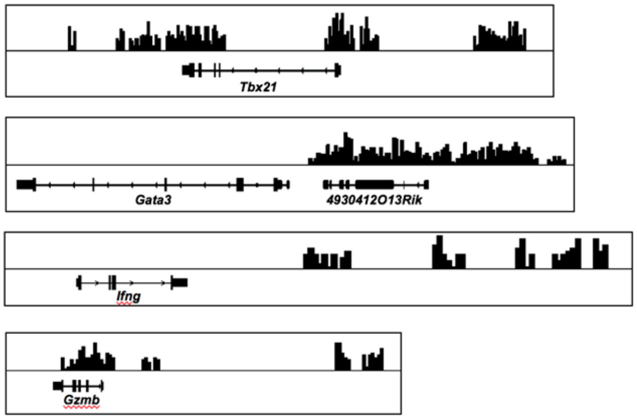


**Figure 2 IGV browser view of H3K27Me3 ChIP-seq peaks at the indicated gene loci in Th17 cells.**

*Corrections in the text*
*In sFig. 2c, STAT vs Stat*
*In Fig 2b and sFig. 2c, I do not see IRF4 as is stated in the text on p7*
*P 16, end of 1st paragraph, s Fig. 7b,c instead of s Fig. 6*
*P 16 2nd paragraph, there is no s Fig. 6 d.*

**Response: Thanks for pointing those annotation mistakes, we have corrected them and included IRF4 in Supplemental Fig. 2c in the revised manuscript.**

*P 8, 2nd chapter. TRIM28 deficiency does not lead to increased Foxp3 expression in Th17 cells. It rather seems to lead to more Treg cells under Th17 conditions, because the IL17+ cells do not have enhanced Foxp3 (Fig. 2a)*
*P 9, 1st chapter. Anti-Il-2 DOES rescue IL17 production by 6-fold (sFig 3d)*

**Response: As the reviewer suggested, we have changed our expression in the revised manuscript.**

*sFig. 3, annotations of b and c are switched, how were pStat molecules measured? FACS? The Figure only shows percentages, not levels as stated.*

**Response: The pSTAT3 was measured by FACS. In response to the reviewer's suggestions, we have corrected the annotation and interpretation in the revised manuscript.**

REVIEWERS' COMMENTS:

Reviewer #2 (Remarks to the Author):

the remaining issues have now been solved.

Reviewer #2

the remaining issues have now been solved.

**Thanks a lot.**
